# A photoresponsive palladium complex of an azopyridyl-triazole ligand: light-controlled solubility drives catalytic activity in the Suzuki coupling reaction†

**DOI:** 10.1039/d1ra03838a

**Published:** 2021-07-09

**Authors:** Attila Kunfi, István Jablonkai, Tamás Gazdag, Péter J. Mayer, Péter Pál Kalapos, Krisztina Németh, Tamás Holczbauer, Gábor London

**Affiliations:** MTA TTK Lendület Functional Organic Materials Research Group, Institute of Organic Chemistry, Research Centre for Natural Sciences Magyar tudósok krt. 2. 1117 Budapest Hungary jablonkai.istvan@ttk.hu london.gabor@ttk.hu; Institute of Chemistry, Eötvös Loránd University Pázmány Péter stny. 1/A 1117 Budapest Hungary; Institute of Chemistry, University of Szeged Rerrich tér 1. 6720 Szeged Hungary; MS Metabolomics Research Group, Instrumentation Center, Research Centre for Natural Sciences Magyar tudósok krt. 2 1117 Budapest Hungary; Centre for Structural Science and Institute of Organic Chemistry, Research Centre for Natural Sciences Magyar tudósok krt. 2. 1117 Budapest Hungary

## Abstract

Herein, the design and synthesis of a click-derived Pd-complex merged with a photoswitchable azobenzene unit is presented. While in the *trans*-form of the switch the complex showed limited solubility, the photogenerated *cis*-form rendered the molecule soluble in polar solvents. This light-controllable solubility was exploited to affect the catalytic activity in the Suzuki coupling reaction. The effect of the substrate and catalyst concentration and light intensity on the proceeding and outcome of the reaction was studied. Dehalogenation of the aryl iodide starting material was found to be a major side reaction; however, its occurrence was dependent on the applied light intensity.

## Abstract 2

A palladium complex of a click-derived pyridyl-triazol ligand with photoswitchable solubility is reported.

## Graphical abstract

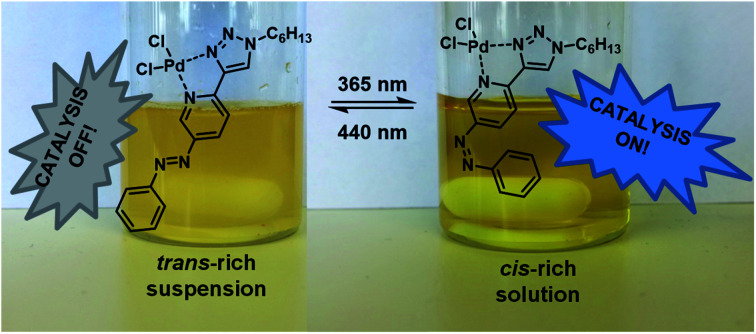

## 1 Introduction

The combination of light-responsive molecular machines with catalytic transformations led to the rapidly developing field of photoswitchable catalysis.^1–4^ Modulating the catalytic activity with light as an input signal can impact the development of catalysis in different ways. Controlling the occurrence of chemical transformations temporally and spatially in molecular systems that approach cellular complexity can be foreseen. Furthermore, the light-induced actuation of different forms of the same catalyst will help us to integrate the aims of green chemistry^5^ such as activity, selectivity and recycling, within a single system through controlled structural changes.^6^ The remarkable progress that has been made in the field of light-driven molecular machines provides a variety of established photochromes to serve as control units.^7^ Among these, azobenzenes, dithienylethenes and overcrowded alkenes are the most frequently exploited units in photoswitchable catalysis. Within the vast set of molecular catalysts, organometallic complexes are arguably the most widely studied systems and used in a myriad of organic transformations. Accordingly, several organometallic complexes with photoswitchable activity have been reported, although the field is still in its infancy.^8,9^ The key feature of these systems is to affect the catalytic event through steric and/or electronic control of the environment around the metal center. Generally, electronic effects are best influenced by dithienylethenes^10–14^ due to changes in the conjugation pattern upon their light-triggered electrocyclization. The levels of steric control are mostly associated with isomerization around double bonds.^15–21^ As the focus in most examples is on the smart incorporation of steric and/or electronic control into the catalyst, more simple possibilities for activity modulation are overlooked.^9^ Among these, a straightforward way to control the occurrence of a reaction could be through manipulating the solubility of the organometallic complex. This type of control could be valuable in cascade reactions, where the insoluble catalyst would stay in a dormant state until UV-light irradiation occurs, upon which the metal-catalysed chemistry would be activated. The Suzuki cross-coupling reaction is among the most used reactions in current organic chemistry; hence, it can be foreseen that these reactions could be part of more complex reaction networks controlled by light-switchable catalysts. However, there are several characteristics of Suzuki coupling such as its heterogeneity (for the solubility of starting materials, inorganic bases are often used), the light absorption of the starting materials and products, and Pd loss *via* Pd black formation, which make its combination with photoswitching processes a challenge. It is probably due to these challenges that cross-coupling reactions in general, Suzuki coupling in particular, are not in the focus of explorations in the context of photoswitchable catalysis. In this contribution, we explored the design of an organometallic catalyst having photoswitchable solubility and its applicability and limitations in the Suzuki coupling reaction.

## 2 Results and discussion

### 2.1 Synthesis and characterization of ligands and Pd complexes

**Fig. 1:**
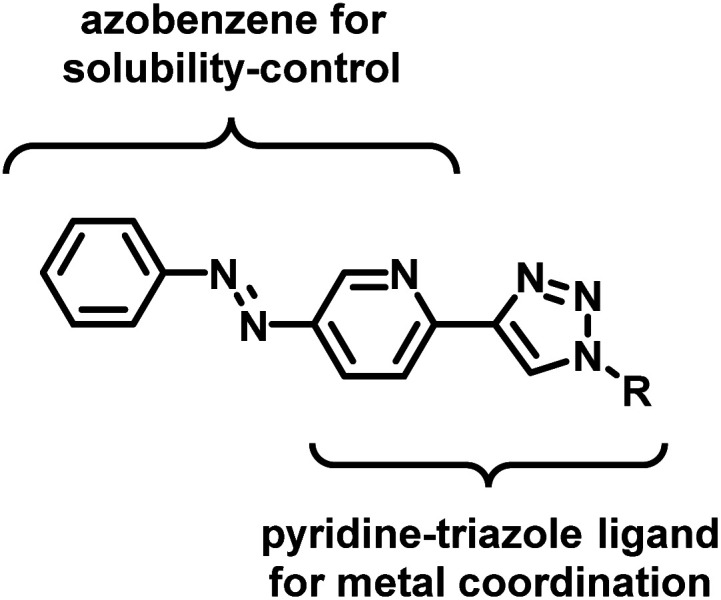
Ligand design for an organometallic catalyst with light-controlled solubility.

In the design of an organometallic catalyst with photocontrollable solubility, several requirements have to be fulfilled. First, a photochromic unit that is responsible for solubility alteration has to be introduced. Second, the light-induced switching of the photochrome has to be preserved in the presence of the coordinating metal. Finally, the complex has to remain stable, without the loss of metal, at the time scale of the process. In our design (Fig. 1), we considered azobenzene^22^ a promising choice of photochrome due to the relatively large change in its dipole moment upon *trans*-to-*cis* isomerization,^23^ which could be associated with different solubilities.^24^ Among the Pd ligands that are suitable for the Suzuki cross-coupling reaction, we turned to the click-derived pyridine–triazole structure,^25–27^ due to its easy synthesis procedure and strong interaction with Pd to ensure homogeneous organometallic catalysis.^28,29^

**Scheme 1:**
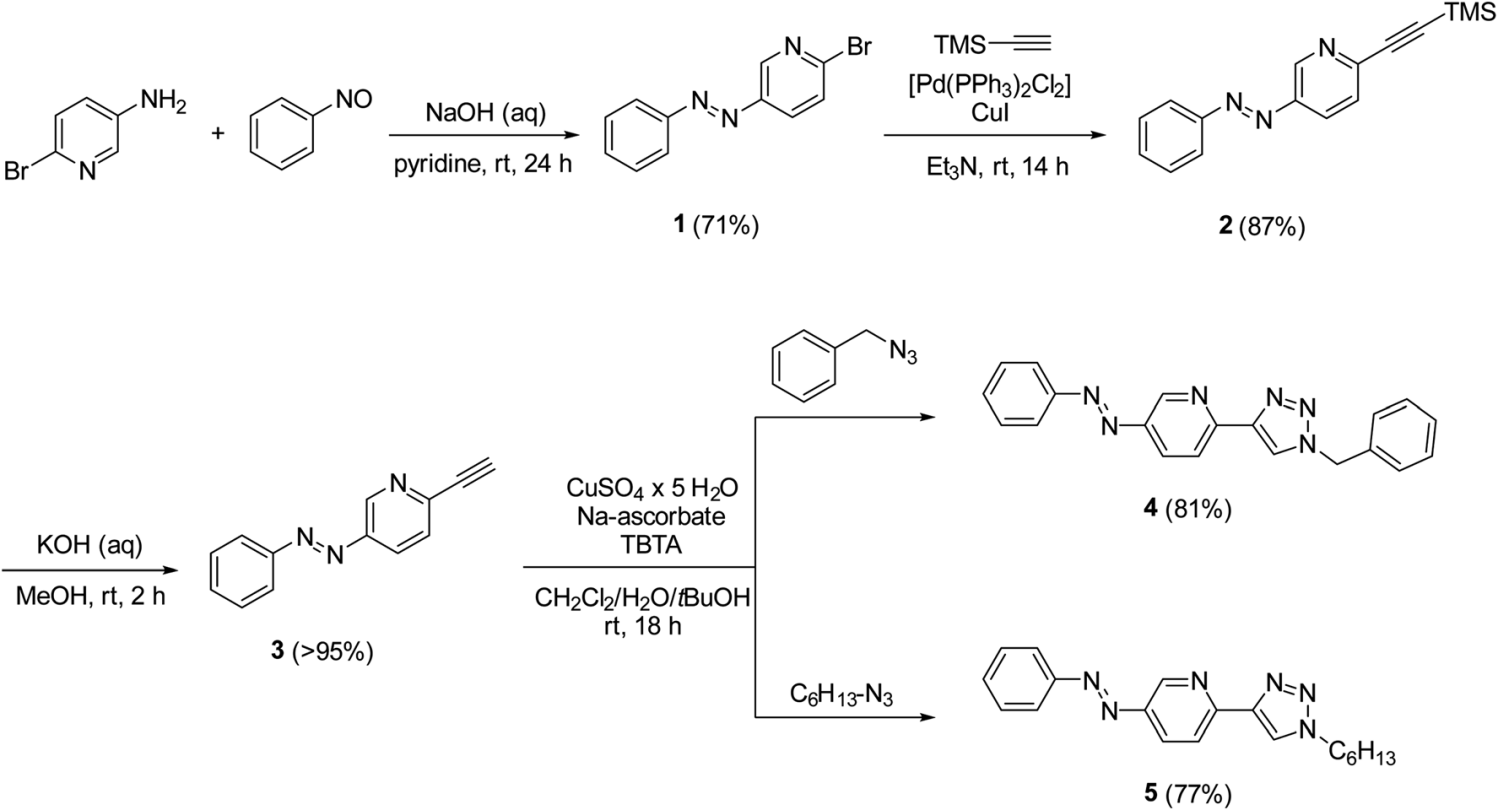
Syntheses of ligands 4 and 5.

To test the hypothesis, we synthesized azobenzene derivatives 4 and 5 with incorporated pyridine-triazole ligands bearing a benzyl and a hexyl side chain, respectively (Scheme 1). The first step of the synthesis was a Mills reaction between 5-amino-2-bromo-pyridine and nitrosobenzene affording 2-bromo-5-(phenylazo)pyridine 1.^30^ A subsequent Sonogashira coupling between 1 and TMS-acetylene followed by the deprotection of alkyne 2 yielded compound 3. The terminal alkyne of compound 3 was transformed into triazole rings *via* azide–alkyne cycloadditions with benzyl azide and hexyl azide leading to ligands 4 and 5, respectively (for detailed synthetic procedures, see ESI†).

**Fig. 2:**
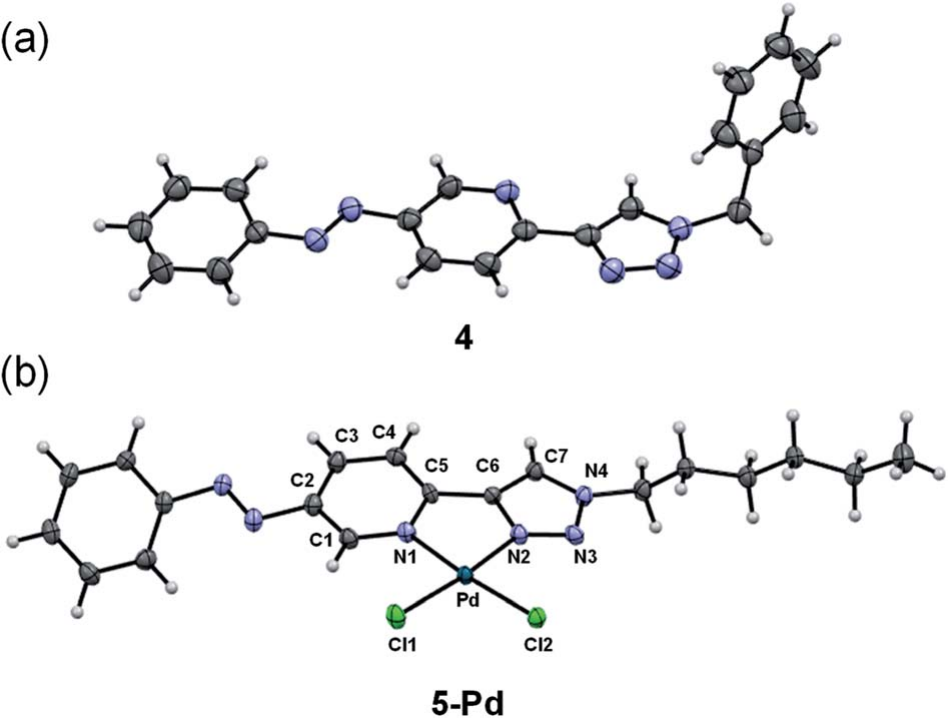
X-ray crystal structures of ligand 4 (a) and complex 5-Pd (b). ORTEP representations are drawn at the 50% probability level.

Ligand 4 could be crystallized for X-ray crystallographic analysis (Fig. 2a), while the crystallization of ligand 5 having a more lipophilic sidechain was unsuccessful.

**Scheme 2:**
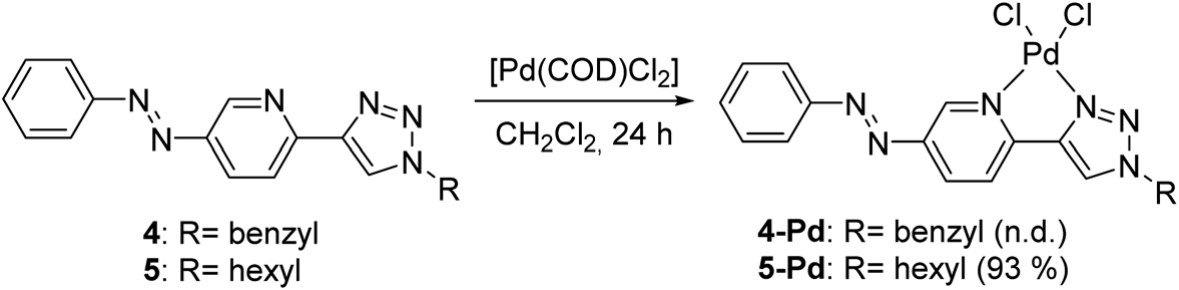
Syntheses of Pd-complexes of ligands 4 and 5.

Both ligands showed reversible photoisomerization in solutions (Fig. S3, ESI†), while no apparent change in their solubility was observed upon irradiation with UV-light (365 nm). Pd-complexes were prepared from ligands 4 and 5 using Pd(COD)Cl_2_ as the metal source (Scheme 2).^25^

In the case of ligand 4 with a benzyl side chain, a yellow solid precipitate was formed that could not be dissolved in any common solvents (solubility was fairly good in DMSO; however, using this solvent led to the loss of coordinated Pd), which prevented its structural characterization. Complex 5-Pd, although precipitated from CH_2_Cl_2_ upon formation, was found to be moderately soluble in common organic solvents. The molecular structure of 5-Pd was confirmed by X-ray crystallography (Fig. 2b). The Pd binds to the ligand moiety through the pyridine nitrogen (N1) and the N2 nitrogen of the triazole ring. The ring N-atoms and the two chlorides provide a square planar coordination mode for the metal. The structure of the pyridine–triazole complex is in agreement with literature examples;^25,28^ hence, the pendant phenylazo group has negligible influence on the metal coordination (Table 1).

**Table 1:** Comparison of selected bond lengths and bond angles of 5-Pd and a related complex (“literature”) previously reported in the literature^25^

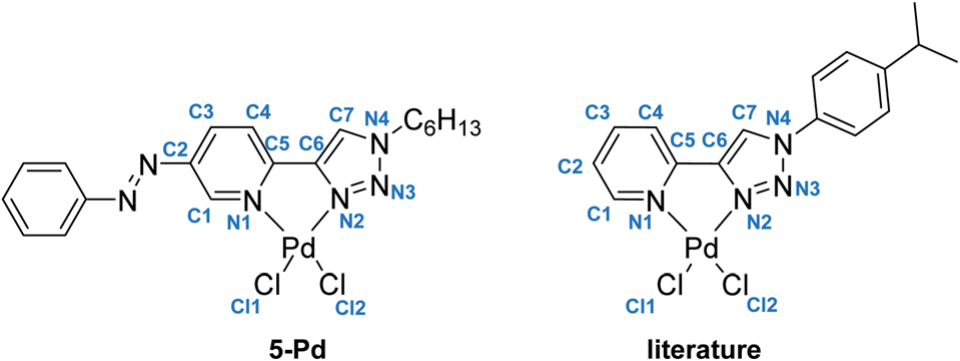					
	Bond length (Å) (5-Pd)	Bond length (Å) (lit)		Bond angle (°) (5-Pd)	Bond angle (°) (lit)
Pd–Cl1	2.285	2.285	N1–Pd–N2	80.26	80.6
Pd–Cl2	2.289	2.264	N1–Pd–Cl1	93.83	94.0
Pd–N1	2.050	2.055	N2–Pd–Cl2	94.79	94.4
Pd–N2	2.005	2.007	Cl1–Pd–Cl2	91.08	91.01
N1–C5	1.353	n.d.			
C5–C6	1.453	1.464			
C6–C7	1.363	1.359			
C7–N4	1.338	1.343			
N4–N3	1.345	1.349			
N3–N2	1.294	1.312			
N2–C6	1.374	1.364			

**Fig. 3:**
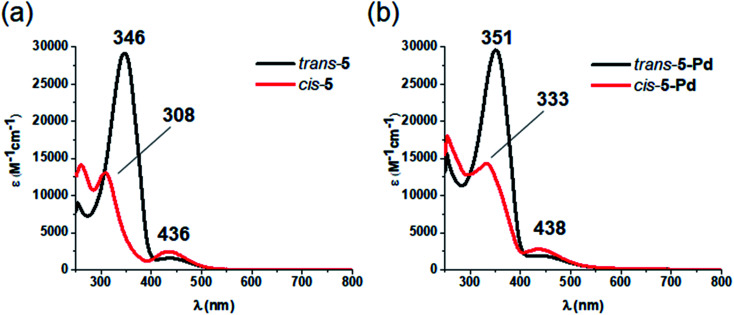
UV-Vis spectra of the *cis*- and *trans*-form of ligand 5 (a) and complex 5-Pd (b) in DMF/H_2_O 1 : 1.

Regarding photochemical properties, no pronounced difference was observed between the free ligand and the complex (Fig. 3). Such minor changes in the absorption properties following Pd complexation were unexpected, but are in agreement with previous reports on Pd and Pt complexes of click-derived ligands.^25^

**Fig. 4:**
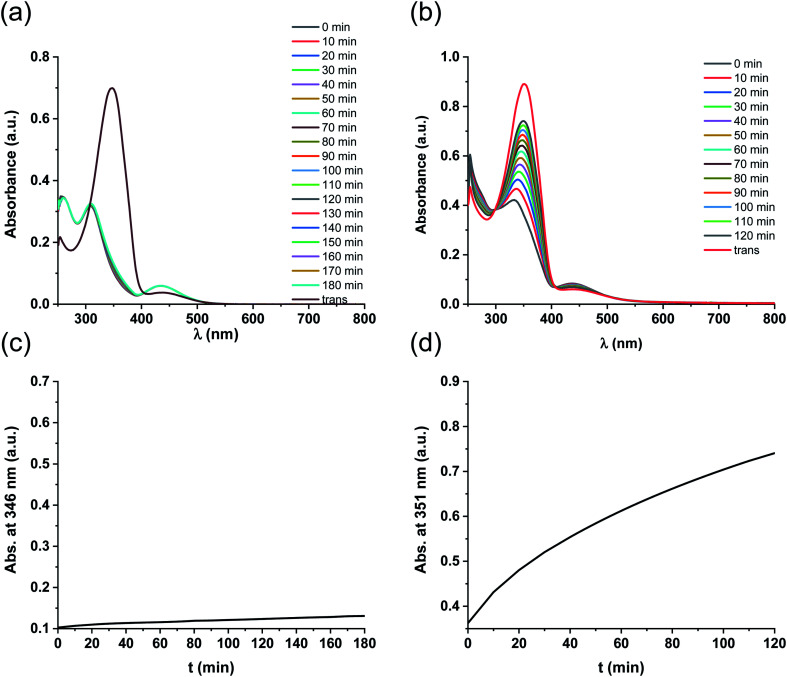
(a) Thermal relaxation of ligand *cis*-5 after its generation from *trans*-5 by irradiation at 365 nm. (b) Thermal relaxation of *cis*-5-Pd after its generation from *trans*-5-Pd by irradiation at 365 nm. (c) Monitoring the thermal relaxation of *cis*-5 at 346 nm. (d) Monitoring the thermal relaxation of *cis*-5-Pd at 351 nm.

Like ligand 5, complex 5-Pd underwent light-induced isomerization to its *cis*-form. The thermal behavior of the photogenerated species, however, was markedly different (Fig. 4) as *cis*-5 exhibited increased stability compared to *cis*-5-Pd. At room temperature, the free ligand showed almost no reverse isomerization in 3 hours (Fig. 4a and c), while most of *cis*-5-Pd isomerized back to its *trans*-form within 2 hours (Fig. 4b and d). This suggests the potential contribution of Pd complexation to a decreased double-bond character of the azo group that leads to lower thermal stability.

**Fig. 5:**
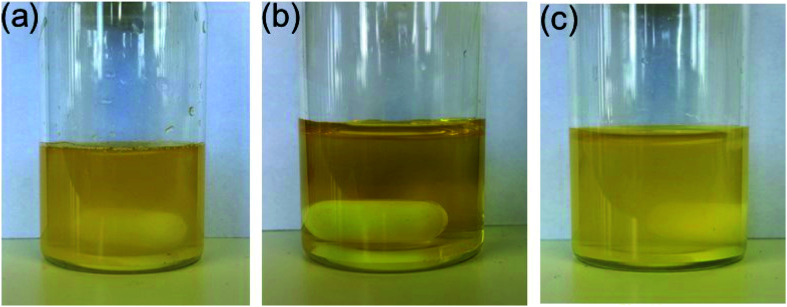
Inhomogeneous mixture of *trans*-5-Pd in DMF/H_2_O 1 : 1 (0.15 mg mL^−1^) (a), which turns into a solution of *cis*-5-Pd upon irradiation with UV-light (365 nm, *P* = 10 W, rt, 30 min) (b); (c) irradiation of the solution with visible light (440 nm, rt, 30 min) results in a cloudy mixture again.

Further experiments revealed that a mixture of DMF/water (1 : 1) was suitable for testing the catalytic activities^28^ of the different isomers in the Suzuki coupling reaction, since *cis*-5-Pd and *trans*-5-Pd showed different solubilities in this solvent system. While *trans*-5-Pd in the solvent mixture (0.15 mg/1 mL) was not fully dissolved forming an inhomogeneous mixture, irradiation of the system with 365 nm light for 20 min led to a clear solution (Fig. 5a and b). Irradiation of the solution with 440 nm light led to the re-formation of the cloudy mixture (Fig. 5c).

**Fig. 6:**
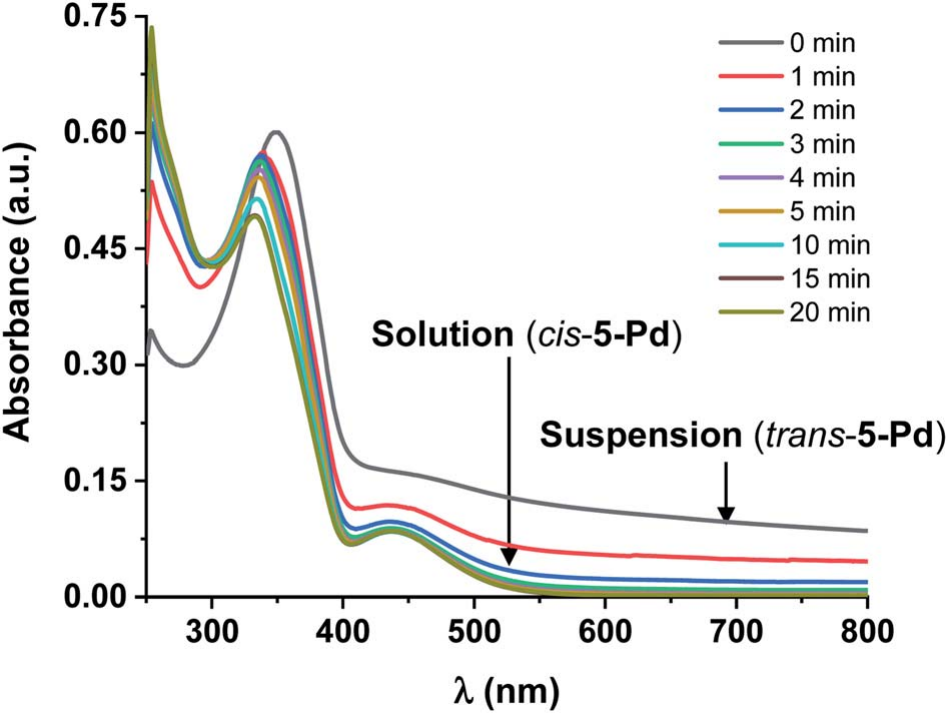
UV-Vis spectra of 5-Pd before and after irradiation at 365 nm in DMF/H_2_O 1 : 1 (*c* = 3 × 10^−4^ M, DMF/H_2_O 1 : 1). The initial spectrum of *trans*-5-Pd reflects the inhomogeneous nature of the systems (low intensity absorption, shifted baseline), which, upon irradiation, is converted to the spectrum of the soluble *cis*-5-Pd.

Further UV-Vis measurements regarding the solubility of the isomers were conducted. Increasing the concentration of *trans*-5-Pd from 10^−5^ M up to 3 × 10^−4^ M in DMF/H_2_O 1 : 1, a cloudy mixture was formed showing a UV-Vis spectrum (Fig. 6) similar to that of *trans*-5-Pd at a lower concentration (Fig. 3). However, it was characterized by lower intensity absorptions and shifted baseline, indicating the inhomogeneity of the sample. Upon irradiation of the suspension, the catalyst was gradually dissolved and after about 20 min a spectrum corresponding to that of *cis*-5-Pd was obtained.

**Fig. 7:**
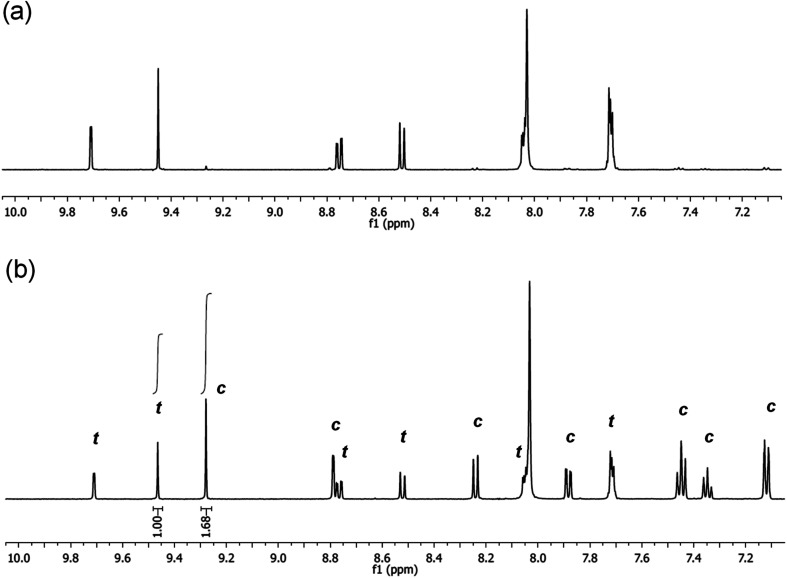
Partial ^1^H NMR spectra (DMF-d7, 500 MHz, 21 °C) of 5-Pd before (a) and after (b) irradiation with UV-light (365 nm, *P* = 10 W, 2 h, rt) (*t* – *trans*-5-Pd, *c* – *cis*-5-Pd).

It is noted, that upon irradiation no apparent Pd black formation was observed, indicating the photostability of 5-Pd. The photostability of the complex was also confirmed by ^1^H NMR spectroscopy (Fig. 7 and S1, ESI†). Upon irradiation of a sample of *trans*-5-Pd for 2 hours, a clean mixture of *trans*-5-Pd and *cis*-5-Pd was formed in a ratio of 1 : 1.7 without any sign of degradation.

### 2.2 Evaluation of catalytic activity

**Fig. 8:**
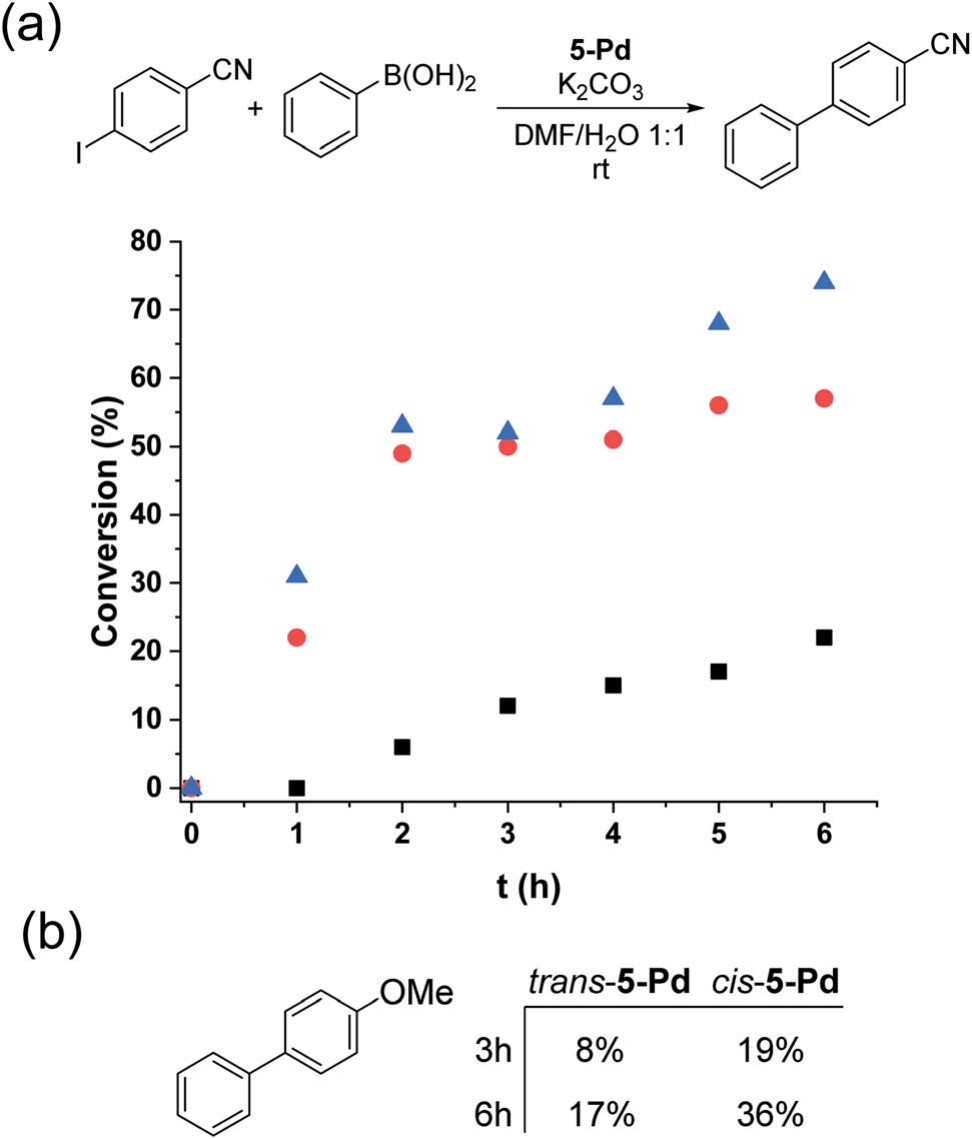
Monitoring the Suzuki coupling reaction of (a) 4-iodobenzonitrile and (b) 4-iodoanisole with phenylboronic acid catalyzed by *trans*- (black squares – 1.5 mol%) and *cis*-5-Pd (red circles – 1.5 mol%, blue triangles – 0.6 mol%). Reaction conditions: phenylboronic acid (0.1 mmol), aryl halide (0.1 mmol), K_2_CO_3_ (0.3 mmol), 5-Pd, DMF/H_2_O 1 : 1 (3 mL), *λ*_max_ = 365 nm, *P* = 10 W, rt.

*Trans*-5-Pd (1.5 mol%) showed low activity in the Suzuki coupling reaction of phenylboronic acid and 4-iodobenzonitrile (1 : 1) that is consistent with its moderate solubility in the reaction medium. After 6 hours in the darkness, only about 20% conversion into 4-cyanobiphenyl was observed (Fig. 8a). In all the reactions, the *cis*-form of the catalyst was generated by the irradiation (*λ*_max_ = 365 nm, *P* = 10 W) of a solution of parent 5-Pd for 30 min at room temperature prior to catalysis. Following catalyst addition, the reaction mixture was continuously irradiated to maintain the concentration of the *cis*-form high. Gratifyingly, when the soluble *cis*-rich 5-Pd (1.5 mol%) was used as a catalyst, a remarkable increase in the activity was observed. In 2 hours, 50% conversion was obtained as compared to 6% by *trans*-5-Pd (Fig. 8a). Furthermore, the catalyst loading could be decreased to 0.6 mol% without any loss of activity.

Time-course studies of the reaction catalyzed by *cis*-5-Pd up to 6 hours revealed that a steep increase in conversion in the first 2 h was followed by a plateau when 1.5 mol% catalyst was used, or a slight further increase up to 70% at a catalyst loading of 0.6 mol% (Fig. 8a). Along with the proceeding of the cross-coupling, the reaction mixture became increasingly dark, indicating gradual loss of metal. Probably, at higher catalyst concentrations, enhanced Pd aggregation led to loss of activity, while at low catalyst loading slower aggregation preserved activity. No such darkening of the reaction mixture was observed in the case of the less active *trans*-5-Pd catalyst. Based on the observed photostability of the complex, the metal loss likely occurs in the catalytic cycle, upon contact with the substrate molecules. Nevertheless, it is clear that the increased catalytic activity of *cis*-5-Pd is strongly dependent on its light-induced dissolution during the reaction allowing a more efficient reaction to take place. Employing 4-iodoanisole in the coupling, similar activity trends of the isomers were found (Fig. 8b).

To exclude a potential photoredox mechanism in the reaction, *cis*-5-Pd was generated in DMF/H_2_O (1 : 1), then the clear catalyst solution was added to the reactants and the mixture was stirred at room temperature in the darkness. Samples were taken after 1 h and 2 h reaction times. After 1 h, 11%, while after 2 h 18%, conversion was obtained. These values are significantly higher than those obtained with *trans*-5-Pd, while much lower than with continuously irradiated reaction mixtures. The lower conversion can be explained with the favoured thermal *cis*-to-*trans* isomerization of the complex leading to catalyst precipitation and hence to the loss of activity.

In an attempt to increase the yield of the Suzuki coupling reaction by reducing the metal loss, the concentration of the starting materials was decreased by 50% (0.05 mmol/3 mL) compared to what was used initially (0.1 mmol/3 mL), while the concentration of the catalyst was kept at 0.6 mol%. This dilution resulted in slower product formation (1 h: 7%; 2 h: 24%) compared to the original conditions (1 h: 31%; 2 h: 53%), while the metal loss was visibly decreased.

**Fig. 9:**
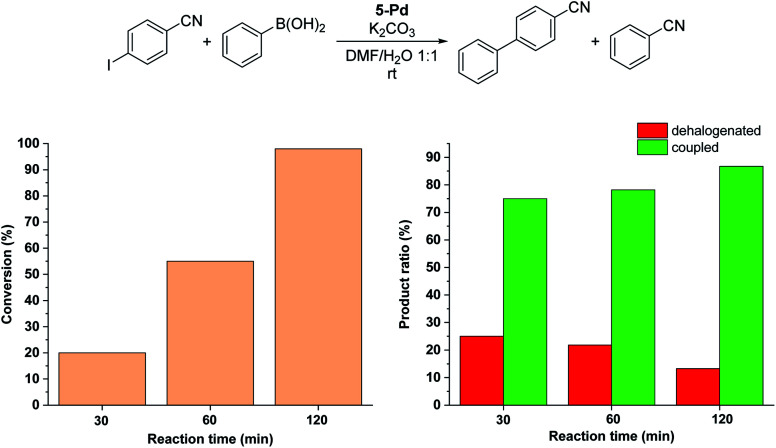
Monitoring the Suzuki coupling reaction of 4-iodobenzonitrile with phenylboronic acid catalyzed by 5-Pd. Reaction conditions: phenylboronic acid (0.05 mmol), 4-iodobenzonitrile (0.05 mmol), K_2_CO_3_ (0.15 mmol), *cis*-5-Pd catalyst solution (DMF/H_2_O 1 : 1, 0.755 mL, 0.6 mol% Pd), DMF/H_2_O 1 : 1 (2.245 mL), *λ*_max_ = 365 nm (continuous irradiation), *P* = 30 W, rt.

Since no considerable Pd loss was observed at lower reactant concentrations, we explored the possibility of increasing the reaction rate by increasing the intensity of the light source from 10 W to 30 W, while keeping the conditions otherwise identical. Gratifyingly, increased light intensity promoted the conversion to completion (Fig. 9), likely due to the shift of the photoequilibrium towards higher *cis*-contents. However, the selectivity of the conversion toward the coupled product somewhat decreased, as the dehalogenation of starting 4-iodobenzonitrile to benzonitrile took place. The light-induced dehalogenation of the aryl iodide was proved by the irradiation of the starting material for 2 h, which yielded benzonitrile (22%). Such photo-induced dehalogenations are precedented in the literature and likely proceed *via* the formation of an aryl radical that abstracts one hydrogen from the solvent.^31–33^

**Fig. 10:**
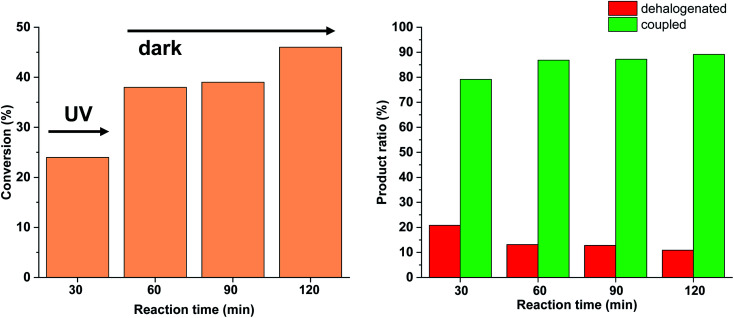
Monitoring the Suzuki coupling reaction of 4-iodobenzonitrile with phenylboronic acid catalyzed by 5-Pd. Reaction conditions: phenylboronic acid (0.05 mmol), 4-iodobenzonitrile (0.05 mmol), K_2_CO_3_ (0.15 mmol), *cis*-5-Pd catalyst solution (DMF/H_2_O 1 : 1, 0.755 mL, 0.6 mol% Pd), DMF/H_2_O 1 : 1 (2.245 mL), rt. Reaction mixture was irradiated at *λ*_max_ = 365 nm (*P* = 30 W) for 30 min, then left in the darkness.

Due to the larger conversions in response to increased light intensity along with decreased selectivity toward the production of the coupled product, we explored how shorter irradiation times would affect the conversion and the product ratio. For this, after the addition of the *cis*-rich 5-Pd solution, the reaction mixture was irradiated for 30 min (30 W), and then the light was switched off for the rest of the reaction time. The conversion profile (Fig. 10) resembled that observed previously under similar conditions with 10 W light source. The conversion was increased initially and then slowed down due to isomerization to the catalytically less active *trans*-5-Pd. When compared to the situation of continuous irradiation (Fig. 9), the conversions were obviously lower when only 30 min irradiation was applied, however, the selectivities did not change substantially (approximately 10–20% dehalogenated and 80–90% coupled in both cases). This suggests that initially dehalogenation takes place more efficiently (most of the dehalogenated product forms in this period), then, after a short initiation period, the Suzuki coupling reaction predominates.

**Fig. 11:**
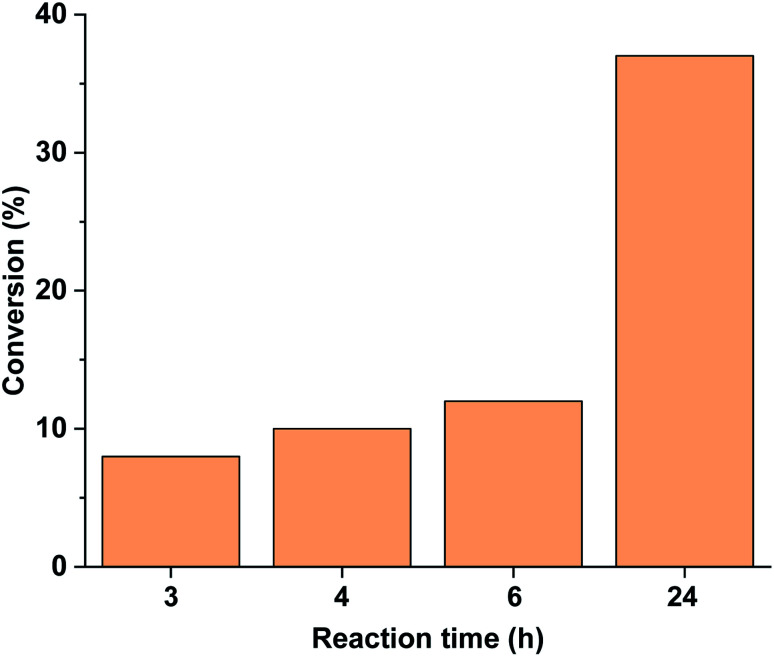
Monitoring the Suzuki coupling reaction of 4-iodobenzonitrile with phenylboronic acid catalyzed by 5-Pd. Reaction conditions: phenylboronic acid (0.05 mmol), 4-iodobenzonitrile (0.05 mmol), K_2_CO_3_ (0.15 mmol), *trans*-5-Pd catalyst suspension (DMF/H_2_O 1 : 1, 0.755 mL, 0.6 mol% Pd), DMF/H_2_O 1 : 1 (2.245 mL), rt, darkness.

Performing the reaction in the darkness using *trans*-5-Pd led to a slow reaction with a conversion of 37% after 24 h (Fig. 11). No dehalogenation was detected in this case. For comparison, the *trans*-5-Pd-catalysed reaction at 40 °C led to 32% conversion (>99% selectivity) after 2 h due to the increased solubility of the catalyst at this temperature. Notably, this conversion is comparable to that obtained with *cis*-5-Pd applying 30 min initial irradiation (Fig. 10), while lower than the conversion upon continuous irradiation of the reaction mixture (Fig. 9).

**Fig. 12:**
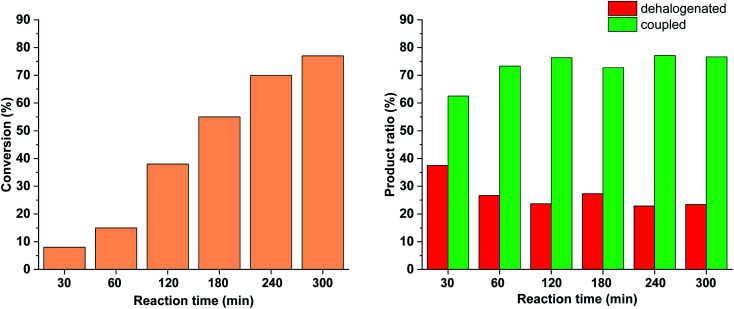
Monitoring the Suzuki coupling reaction of 4-iodobenzonitrile with phenylboronic acid catalyzed by 5-Pd. Reaction conditions: phenylboronic acid (0.05 mmol), 4-iodobenzonitrile (0.05 mmol), K_2_CO_3_ (0.15 mmol), *cis*-5-Pd catalyst solution (DMF/H_2_O 1 : 1, 0.755 mL, 0.6 mol% Pd), DMF/H_2_O 1 : 1 (2.245 mL), *λ*_max_ = 365 nm (continuous irradiation), *P* = 15 W, rt.

To further tune the conditions towards selective and high-yielding Suzuki coupling, the light intensity was decreased to 15 W (Fig. 12). Compared to the continuous irradiation with 30 W light, in this case the reaction rate, the conversion and the selectivity toward the Suzuki product were lower. Probably, the slower Suzuki reaction due to lower amount of *cis*-5-Pd facilitated the formation of an increased amount of side products. As previously (Fig. 9 and 10), the relative amount of the dehalogenated side product exhibited maximum at the early stage of the reaction.

**Fig. 13:**
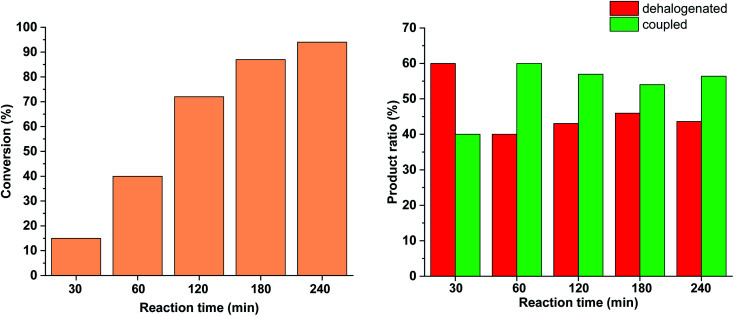
Monitoring the Suzuki coupling reaction of 4-iodobenzonitrile with phenylboronic acid catalyzed by 5-Pd. Reaction conditions: phenylboronic acid (0.05 mmol), 4-iodobenzonitrile (0.05 mmol), K_2_CO_3_ (0.15 mmol), *cis*-5-Pd catalyst solution (DMF/H_2_O 1 : 1, 0.378 mL, 0.3 mol% Pd), DMF/H_2_O 1 : 1 (2.622 mL), *λ*_max_ = 365 nm (continuous irradiation), *P* = 30 W, rt.

Similar effect was observed when the catalyst concentration was decreased to 0.3 mol% keeping the light intensity at 30 W (Fig. 13). At the reduced catalyst concentration, regardless of the ratio of photogenerated *cis*-5-Pd, the rate of the Suzuki coupling step slowed down allowing the formation of an increased amount of dehalogenated products.

Based on the above-mentioned measurements it is clear that the rate of dehalogenation is highest under continuous irradiation of the reaction mixture with 30 W light intensity, while the activity of *cis*-5-Pd in this side reaction is negligible (Fig. 10). Furthermore, higher amount of *cis*-5-Pd can be generated using 30 W light, which is active in Suzuki coupling even in the darkness until back isomerization takes place (Fig. 10). Combining these observations, a feasible approach to facilitate Suzuki coupling along with suppressed dehalogenation could be the occasional switching of the catalyst under the reaction conditions. Regular, relatively short-time irradiations of the mixture could keep the catalyst mainly in its *cis*-form, while the dehalogenation would be somewhat less pronounced due to the non-continuous irradiation.

**Fig. 14:**
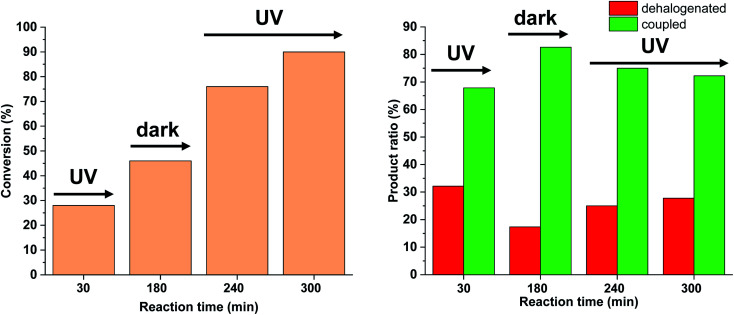
Monitoring the Suzuki coupling reaction of 4-iodobenzonitrile with phenylboronic acid catalyzed by 5-Pd. Reaction conditions: phenylboronic acid (0.05 mmol), 4-iodobenzonitrile (0.05 mmol), K_2_CO_3_ (0.15 mmol), *cis*-5-Pd catalyst solution (DMF/H_2_O 1 : 1, 0.755 mL, 0.6 mol% Pd), DMF/H_2_O 1 : 1 (2.245 mL), *λ*_max_ = 365 nm, *P* = 30 W, rt.

We tested this hypothesis by irradiating the reaction mixture initially for 30 min, and then turned off the light for the next 150 min (Fig. 14). As previously, under continuous irradiation in the first 30 min, we observed the formation of both products. However, when the light was switched off, as expected, the Suzuki coupling reaction was the primary transformation, while the amount of the dehalogenated product did not increase. Turning on the light again, the amount of both products was increased leading to decreased selectivity towards the coupling product.

**Fig. 15:**
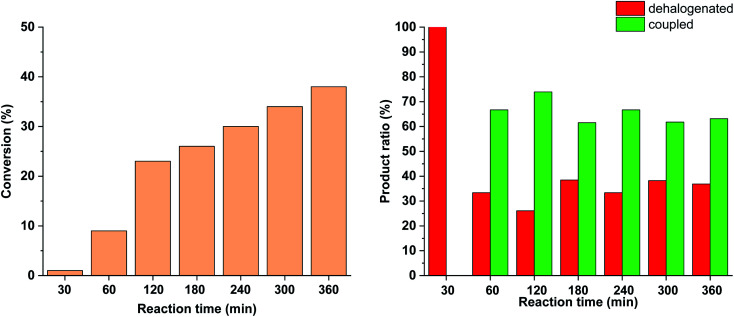
Monitoring the Suzuki coupling reaction of 4-iodoanisole with phenylboronic acid catalyzed by 5-Pd. Reaction conditions: phenylboronic acid (0.05 mmol), 4-iodoanisole (0.05 mmol), K_2_CO_3_ (0.15 mmol), *cis*-5-Pd catalyst solution (DMF/H_2_O 1 : 1, 0.755 mL, 0.6 mol% Pd), DMF/H_2_O 1 : 1 (2.245 mL), *λ*_max_ = 365 nm (continuous irradiation), *P* = 30 W.

We tested the coupling of 4-iodoanisole with phenylboronic acid under irradiation using a 30 W light source and lower initial reactant concentrations (Fig. 15). Compared to the initial conditions (Fig. 8b), higher conversions were obtained; however, as in the case of the reaction of 4-iodobenzonitrile, a large amount of dehalogenated product was detected.

Apart from aryl iodides, we attempted the coupling of aryl bromides (4-bromoacetophenone, 4-bromonitrobenzene) with phenylboronic acid. Unfortunately, these substrates showed no reactivity towards the Suzuki product in the presence of 5-Pd, and only the photodehalogenation products were obtained under continuous irradiation.

## 3 Discussion

The above-mentioned experimental results point towards the feasibility of impacting catalytic activity in the Suzuki cross-coupling reaction by photoswitching catalyst solubility. However, care should be taken if one considers integrating this chemistry into more complex systems, such as cascade transformations or reaction networks. Light intensity seems to be an important factor in faster/more complete photoswitching between the two forms of the catalyst. Increased light intensity, in the case of the model Suzuki reaction, led to higher conversions and faster response times, with the downside of initiating a considerable amount of side products through photodehalogenation. This latter problem could be a general disadvantage in coupling reactions beyond the Suzuki reaction. The amount of product and side product was found to be dependent also on the catalyst/substrate ratio. It is also clear that a faster Suzuki reaction (electron poor 4-iodobenzonitrile) is preferred if better selectivities are targeted, as photodehalogenation takes place mainly in the initial stage of the reaction. With further catalyst engineering, the light-induced side reaction could be eliminated if a molecule with thermally very stable and soluble *cis*-form could be synthesized (note that the impact of the reaction components on the stability of the *cis*-form is unknown). However, the soluble, catalytically active, but thermally less stable *cis*-catalyst could be exploited in dissipative molecular systems.^34,35^

It is important to note that although tuning product formation in the Suzuki reaction *via* photoswitching solubility is demonstrated through this work, the expansion of this control to a wide substrate scope is not trivial. Solubility control is demonstrated in DMF/H_2_O 1 : 1 mixture, which might not be an ideal solvent mixture for many substrates due to their limited solubility. This is illustrated by the lack of conversion in the case of 4-iodonitrobenzene, due to its poor solubility in DMF/H_2_O 1 : 1. Moreover, the light absorption of several substrates/coupled products might interfere with the irradiation wavelength that decreases (or even eliminates) the possibility of catalyst isomerization. Hence, expanding the substrate scope could lead to individual optimization of conditions (and catalysts) for each starting material. The same holds for expanding the scope of cross-coupling reactions where light-controlled solubility can be exploited in controlling the product formation. Our attempt to use 5-Pd in Sonogashira coupling remained inconclusive. Not only aqueous Sonogashira couplings are challenging, but also the low solubility of acetylenes and the additional components (CuI) in the reaction mixture did not facilitate the catalytic study.

## 4 Conclusion

In summary, we have designed and synthesized an azobenzene-containing Pd-complex with photocontrollable solubility, which was exploited in the light-enhanced Suzuki cross-coupling reaction. Our design of a photostable Pd complex with switchable solubility could open new ways in the development of photoswitchable organometallic catalysts and catalytic systems with increased complexity. However, care should be taken in choosing the components (solvent, base, starting materials) and conditions (light intensity) of the coupling reaction to avoid the occurrence of unwanted processes (metal loss or dehalogenation) that might influence the outcome or hinder parallel reactions.

## Conflicts of interest

There are no conflicts of interest to declare.

## Supplementary Material

RA-011-D1RA03838A-s001

RA-011-D1RA03838A-s002
