# Supplementary material for: A photoresponsive palladium complex of an azopyridyl-triazole ligand: light-controlled solubility drives catalytic activity in the Suzuki coupling reaction
Source: RSC Adv. 2021 Jul 9;11(38):23419–29. doi: 10.1039/d1ra03838a (PMC9036612; doi:10.1039/d1ra03838a)
Supplement: RA-011-D1RA03838A-s001 [file RA-011-D1RA03838A-s001.pdf]

Electronic Supplementary Material (ESI) for RSC Advances.
This journal is © The Royal Society of Chemistry 2021

Supporting Information

Photoresponsive palladium complex of an azopyridyl-triazole ligand: light-controlled solubility drives catalytic activity in Suzuki-coupling reaction

Attila Kunfi,[a] István Jablonkai,*[a] Tamás Gazdag,[a,b] Péter J. Mayer,[a,c] Péter Pál Kalapos,[a] Krisztina Németh,[b,d] Tamás Holczbauer,[e] and Gábor London*[a]

[a] MTA TTK Lendület Functional Organic Materials Research Group, Institute of Organic Chemistry, Research Centre for Natural Sciences, 1117 Budapest, Magyar tudósok krt. 2., Hungary; Emails: jablonkai.istvan@ttk.hu (I.J.), london.gabor@ttk.hu (G.L.)
[b] Institute of Chemistry, Eötvös Loránd University, Pázmány Péter stny. 1/A, 1117, Budapest, Hungary
[c] Institute of Chemistry, University of Szeged, 6720 Szeged, Rerrich tér 1., Hungary
[d] MS Metabolomics Research Group, Instrumentation Center, Research Centre for Natural Sciences, 1117 Budapest, Magyar tudósok krt. 2, Hungary
[e] Institute of Organic Chemistry and Centre for Structural Science, Research Centre for Natural Sciences, 1117 Budapest, Magyar tudósok krt. 2., Hungary

Table of contents

1. General remarks 2
1. Synthesis 3
2. UV-Vis data 10
3. X-Ray Crystallography 11
5. NMR Spectra 17
6. References 23

1. General remarks

Commercial reagents, solvents and catalysts were purchased (Aldrich, Fluorochem, VWR) as reagent–grade and used without further purification. Solvents for extraction were of technical quality and were distilled for column chromatography. For spectroscopy and sample treatment opti-grade quality solvents were used. Organic solutions were concentrated by rotary evaporation at 25-40 °C. Thin layer chromatography was carried out on SiO_2–layered aluminum plates (60778-25EA, Fluka). Column chromatography was performed using SiO_2–60 (230–400 mesh ASTM, 0.040–0.063 mm from Merck) at 25 °C or using a Teledyne Isco CombiFlash® Rf+ automated flash chromatographer with silica gel (25-40 µm, Redisep Gold®). Room temperature refers to 23(+/- 0.5)°C.

NMR spectra were acquired on a Varian 500 NMR spectrometer, running at 500 and 126 MHz for ^1H and ^{13}C, respectively, and on a Varian 300 NMR spectrometer, running at 300 and 75 MHz for ^1H and ^{13}C, respectively. NMR spectrometers were operated at 30°C, otherwise noted. The residual solvent peaks were used as the internal reference. Chemical shifts (δ) are reported in ppm. The following abbreviations are used to indicate the multiplicity in ^1H NMR spectra: s, singlet; d, doublet; t, triplet; q, quartet; p, pentett; m, multiplet. ^{13}C NMR spectra were acquired on a broad band decoupled mode.

UV-Vis spectra were measured with a Jasco V-750 spectrophotometer. Data were collected from 800 nm to 200 nm using 1 nm data interval, 2 nm bandwidth, and 400 nm/s scan speed.

LC-MS analyses of several intermediates were performed on a Shimadzu LCMS-2020 System operated in electrospray ionization mode.

GC-MS analysis of product mixtures were performed on a Shimadzu GCMS-QP2010 Ultra System operated in electron impact ionization (EI) mode.

High resolution measurements were performed on a Sciex TripleTOF 5600+ high resolution tandem mass spectrometer equipped with DuoSpray ion source. Electrospray ionization was applied in positive ion detection mode. Samples were dissolved in acetonitrile and flow injected into acetonitrile/water 1:1 flow, or otherwise noted. The flow rate was 0.2 mL/min. The resolution of the mass spectrometer was 35000.

Irradiation of samples were carried out with a 365 nm and 440 nm 10 W COB LED, or in a custom-built 30 W photoreactor operated at a current of 1.0 A supported by a Voltcraft PPS-

11603 power supply. The 30 W photoreactor was equipped with a continuous-flow cooling system (Figure S2, ESI). 15 W input power was achieved with the 30 W LED operated at 0.5 A.

1. Synthesis

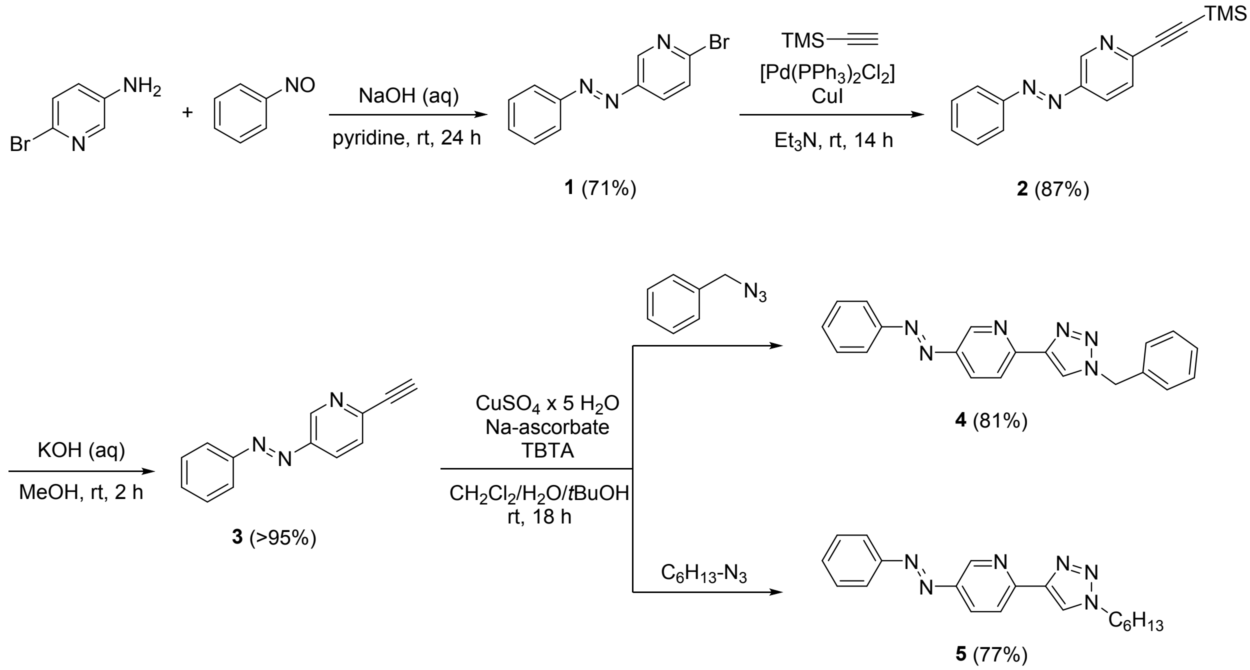

Scheme S1. Synthesis of ligands **4** and **5**.

(*E*)-2-bromo-5-(phenyldiazenyl)pyridine (1)[1]

To a solution of 5-amino-2-bromo-pyridine (1.43 g, 8.3 mmol) and nitrosobenzene (0.9 g, 8.4 mmol) in pyridine (60 mL) was added a solution of NaOH (336 mg in 2 mL water). The mixture was stirred for 24 h at rt. The solvent was removed *in vacuo*, the residue was dissolved in ethyl acetate (60 mL) and washed with water (2x30 mL). The organic phase was dried ($MgSO_4$), filtered, and the solvent was evaporated under reduced pressure. The residue was purified by silica gel chromatography using gradient elution (hexanes to hexane-ethyl acetate 20%) to obtain an orange-coloured product (1.54 g, 71%). ^{1}H NMR (500 MHz, $CDCl_3$) δ = 8.95 (d, J = 2.5 Hz, 1H), 8.01 (dd, J = 8.5, 2.6 Hz, 1H), 7.92 – 7.95 (m, 2H), 7.61 (d, J = 8.5 Hz, 1H), 7.51 – 7.56 (m, 3H) ppm; ^{13}C NMR (126 MHz, $CDCl_3$) δ = 152.53, 148.08, 147.27, 144.15, 132.21, 129.39 (2C), 129.14, 128.78, 123.32 (2C) ppm. HRMS (ESI) calcd for $C_{11}H_9N_3Br$ 261.9974 [MH^+], found 261.9977.

(*E*)-5-(phenyldiazenyl)-2-((trimethylsilyl)ethynyl)pyridine (2)

A mixture of **1** (500 mg, 2 mmol), trimethylsilyl acetylene (235 mg, 2.4 mmol), $Pd(PPh_3)_2Cl_2$ (46 mg, 0.065 mmol), CuI (24 mg, 0.125 mmol) and Et_3N (8 mL) was stirred at room temperature for 14 h. After the removal of Et_3N by rotary evaporation the residual oil was dissolved in dichloromethane (30 mL) and washed with sat. NH_4Cl (2x20 mL). The organic phase was dried over $MgSO_4$, filtered, and the solvent was evaporated under reduced pressure. The residue was purified by silica gel chromatography using gradient elution (hexanes to hexane-ethyl acetate 20%) to obtain the orange-coloured crystalline product **2** (485 mg, 87%). ^{1}H NMR (500 MHz, $CDCl_3$) δ = 9.15 (d, *J* = 1.9 Hz, 1H), 8.09 (dd, *J* = 8.4, 2.4 Hz, 1H), 7.93 ($dd_{apparent}$, *J* = 7.9, 1.7 Hz, 2H), 7.57 (d, *J* = 8.0 Hz, 1H), 7.48 – 7.54 (m, 3H), 0.30 (s, 9H) ppm; ^{13}C NMR (126 MHz, $CDCl_3$) δ = 152.69, 147.93, 146.66, 144.90, 132.05, 129.33 (2C), 127.81, 126.88, 123.28 (2C), 103.70, 97.66, -0.18 (3C) ppm. HRMS (ESI) calcd for $C_{16}H_{18}N_3Si$ 280.1264 [MH^+], found 280.1264.

(*E*)-5-(phenyldiazenyl)-2-(prop-1-yn-1-yl)pyridine (3)

To a solution of **2** (480 mg, 1.72 mmol) in methanol (22 mL) was added 1N KOH (4.28 mL) during stirring. After 2 h the solution was diluted with water (80 mL) and extracted with ethyl acetate (2x50 mL). The organic phase was dried over $MgSO_4$, filtered, and the solvent was evaporated under reduced pressure. The resulting product (yellow/orange solid) was sufficiently clean (contains the *cis*-isomer as impurity) to use in the next reaction without further purification. ^{1}H NMR (500 MHz, $CDCl_3$) δ = 9.16 (d, *J* = 2.2 Hz, 1H), 8.11 (dd, *J* = 8.3, 2.4 Hz, 1H), 7.94 ($dd_{apparent}$, *J* = 7.8, 1.9 Hz, 2H), 7.61 (d, *J* = 8.3 Hz, 1H), 7.50 – 7.55 (m, 3H), 3.30 (s, 1H) ppm; ^{13}C NMR (126 MHz, $CDCl_3$) δ = 152.65, 147.76, 146.97, 144.11, 132.17, 129.36 (2C), 128.01, 127.16, 123.32 (2C), 82.79, 79.30. HRMS(ESI) calcd for $C_{13}H_{10}N_3$ 208.0869 [MH^+], found 208.0872.

(*E*)-2-(1-benzyl-1H-1,2,3-triazol-4-yl)-5-(phenyldiazenyl)pyridine (4)

A mixture of **3** (104 mg, 0.5 mmol), benzylazide (62.5 µL, 0.5 mmol), $CuSO_4$ x 5 H_2O (6.5 mg, 25 µmol), sodium L-ascorbate (20 mg, 0.1 mmol) and tris((1-benzyl-4-triazolyl)methyl)amine (TBTA) (5 mg, 9.4 µmol) in CH_2Cl_2-H_2O-^{t}BuOH (1:2:1, 5 mL) was vigorously stirred overnight at room temperature. The reaction mixture was diluted with dichloromethane (20 mL) and

washed with water (2x10 mL). The organic layer was dried over $MgSO_4$, filtered, and the solvent was evaporated under reduced pressure. The orange crystalline product **4** (138 mg, 81%) was isolated by silica gel chromatography using hexane-ethyl acetate gradient elution (10% to 30% v/v). ^{1}H NMR (500 MHz, $CDCl_3$) δ = 9.13 (s, 1H), 8.34 (d, J = 8.5 Hz, 1H), 8.25 (dd, J = 8.5, 2.0 Hz, 1H), 8.15 (s, 1H), 7.95 ($d_{apparent}$, J = 6.9 Hz, 2H), 7.50 – 7.55 (m, 3H), 7.35 – 7.42 (m, 5H), 5.61 (s, 2H) ppm; ^{13}C NMR (126 MHz, $CDCl_3$) δ = 152.79, 147.87, 134.39, 131.79, 129.40 (2C), 129.34 (2C), 129.11, 128.51 (2C), 127.67, 123.21 (2C), 122.88, 120.72, 54.66 ppm. HRMS(ESI) calcd for $C_{20}H_{17}N_6$ 341.1509 [MH^+], found 341.1508.

(*E*)-2-(1-hexyl-1H-1,2,3-triazol-4-yl)-5-(phenyldiazenyl)pyridine (5)

A mixture of **3** (104 mg, 0.5 mmol), 1-hexylazide (64 mg, 0.5 mmol), $CuSO_4$ x 5 H_2O (6.5 mg, 25 μmol), sodium L-ascorbate (20 mg, 0.1 mmol) and tris((1-benzyl-4-triazolyl)methyl)amine (TBTA) (2.7 mg, 5 μmol) in CH_2Cl_2-H_2O-^tBuOH (1:2:1, 5 mL) was vigorously stirred overnight at room temperature. The reaction mixture was diluted with dichloromethane (20 mL) and washed with water (2x10 mL). The organic layer was dried over $MgSO_4$, filtered, and the solvent was evaporated under reduced pressure. The orange crystalline product **5** (129 mg, 77%) was isolated by silica gel chromatography using hexane-ethyl acetate gradient elution (10% to 30% v/v). ^{1}H NMR (500 MHz, $CDCl_3$) δ = 9.17 (d, J = 2.0 Hz, 1H), 8.33 (d, J = 8.5 Hz, 1H), 8.25 (dd, J = 8.5, 2.3 Hz, 1H), 8.22 (s, 1H), 7.95 ($d_{apparent}$, J = 6.9 Hz, 2H), 7.49 – 7.55 (m, 3H), 4.44 (t, J = 7.2 Hz, 2H), 1.98 ($quint_{apparent}$, J = 5.0 Hz, 2H), 1.30 – 1.40 (m, 6H), 0.89 (t, J = 7.0 Hz, 3H) ppm; ^{13}C NMR (126 MHz, $CDCl_3$) δ = 152.80, 152.45, 148.00, 147.96, 147.25, 131.73, 129.32 (2C), 127.60, 123.19 (2C), 122.69, 120.63, 50.75, 31.30, 30.35, 26.29, 22.55, 14.07 ppm. HRMS (ESI) calcd for $C_{19}H_{23}N_6$ 335.1978 [MH^+], found 335.1988.

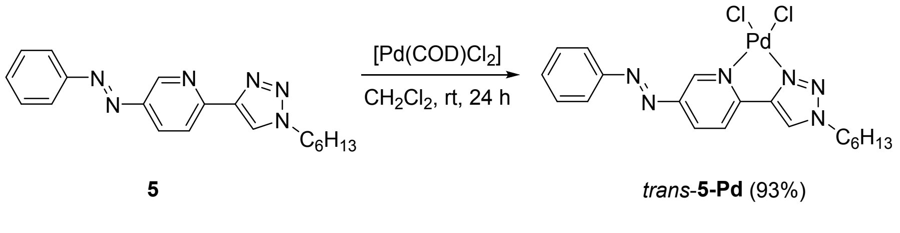

Scheme S2. Synthesis of *trans*-**5-Pd** catalyst.

Preparation of *trans*-5-Pd

To a solution of ligand **5** (33.4 mg, 0.1 mmol) in dichloromethane (3.5 mL) was added dichloro(1,5-cyclooctadiene)palladium (28.5 mg, 0.1 mmol) and the initially homogenous solution was stirred for 1 day. The precipitated palladium complex was filtered off and washed with diethyl ether to obtain the pale orange product *trans*-**5-Pd** (48 mg, 93%). ^{1}H NMR (500 MHz, DMF-d7) δ = 9.71 (d, J = 1.9 Hz, 1H), 9.45 (s, 1H), 8.75 (dd, J = 8.5, 2.2 Hz, 1H), 8.51 (d, J = 8.4 Hz, 1H), 8.05 – 8.03 (m, 2H), 7.72 – 7.69 (m, 3H), 4.74 (t, J = 7.3 Hz, 2H), 2.04 (quint$_{apparent}$, J = 5.0 Hz, 2H), 1.45 – 1.30 (m, 6H), 0.89 (t, J = 7.0 Hz, 3H) ppm; ^{13}C NMR (126 MHz, DMF-d7) δ = 153.40, 151.42, 148.63, 148.35, 147.44, 134.27, 133.44, 130.86, 127.55, 124.51, 123.89, 53.75, 32.07, 30.61, 26.78, 23.29, 14.57 ppm.

HRMS measurements of complex **5-Pd** indicated the loss of HCl from the molecule and subsequent solvent coordination under the measurement conditions (upon ionization). Results obtained from measurements in different solvents to support the ligand exchange process are shown in Table S1. In all cases, the sample solutions were directly injected in the MS detector. Note that in Table S1 M = $C_{19}H_{21}N_6ClPd$ [$M_{\mathbf{5\text{-}Pd}}$ – HCl], 474.0551.

Table S1. HRMS data of **5-Pd** measured in different solvents (M= $C_{19}H_{21}N_6ClPd$ [$M_{\mathbf{5\text{-}Pd}}$ – HCl], 474.0551).

Solvent	Found mass $[M+H]^+$	Formula $[M+H]^+$	Calcd mass $[M+H]^+$	Error / mDa	Error / ppm	Composition
**MeOH**	475.0626	$C_{19}H_{22}N_6ClPd$	475.0629	-0.3226	-0.6791	$[M + H]^+$
	492.0869	$C_{19}H_{25}N_7ClPd$	492.0894	-2.5717	-5.2262	$[M + NH_4]^+$
	507.0882	$C_{20}H_{26}N_6OClPd$	507.0891	-0.8374	-1.6515	$[M + H + CH_3OH]^+$
	516.0896	$C_{21}H_{25}N_7ClPd$	516.0894	0.1282	0.2484	$[M + H + CH_3CN]^+$
**EtOH**	475.0619	$C_{19}H_{22}N_6ClPd$	475.0629	-1.0226	-2.1526	$[M + H]^+$
	492.0885	$C_{19}H_{25}N_7ClPd$	492.0894	-0.9717	-1.9748	$[M + NH_4]^+$
	516.0889	$C_{21}H_{25}N_7ClPd$	516.0894	-0.5717	-1.1079	$[M + H + CH_3CN]^+$
	520.1009	$C_{21}H_{27}N_6OClPd$	520.0969	3.9375	7.5706	$[M + H + CH_3CH_2O]^+$
**CH_3CN**	475.0623	$C_{19}H_{22}N_6ClPd$	475.0629	-0.6226	-1.3106	$[M + H]^+$
	492.089	$C_{19}H_{25}N_7ClPd$	492.0894	-0.4717	-0.9587	$[M + NH_4]^+$
	516.0889	$C_{21}H_{25}N_7ClPd$	516.0894	-0.5717	-1.1079	$[M + H + CH_3CN]^+$
**CD_3CN**	492.0889	$C_{19}H_{25}N_7ClPd$	492.0894	-0.5717	-1.1619	$[M + NH_4]^+$
	519.1079	$C_{21}\ H_{22}D_3N_7ClPd$	519.1083	-0.402	-0.7744	$[M + H + CD_3CN]^+$

Irradiation experiment to generate *cis*-5-Pd

Trans-**5-Pd** (~ 2 mg) was dissolved in DMF-d7 and the sample was irradiated for 2 h at rt and the ^{1}H NMR (500 MHz) spectra of the resulting mixture was recorded at 21°C (Figure S1). No darkening of the solution was observed during irradiation, which indicates the lack of metal loss.

^{1}H NMR (500 MHz, DMF-d7) δ= 9.28 (s, 1H), 8.79 (d, *J* = 1.8 Hz, 1H), 8.24 (d, *J* = 8.5 Hz, 1H), 7.88 (dd, *J* = 8.4, 2.2 Hz, 1H), 7.45 (t, *J* = 7.8 Hz, 2H), 7.35 (z, *J* = 7.4 Hz, 1H), 7.12 (m, 2H), 4.67 (t, *J* = 7.2 Hz, 2H), 1.98 (q, *J* = 7.5 Hz, 2H). Further peaks in the alkyl region cannot be resolved due to overlap with remaining starting material, *trans*-**5-Pd**.

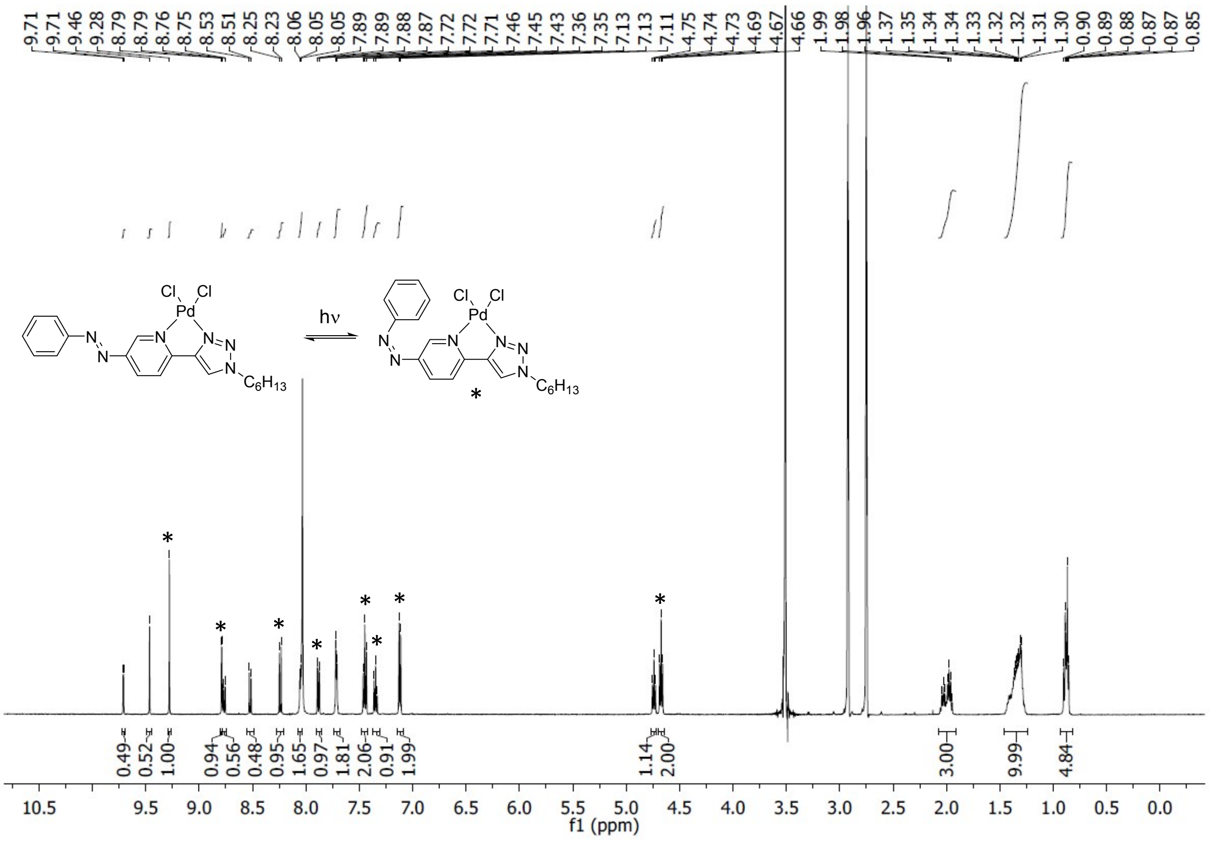

Figure S1. Irradiation of *trans*-**5-Pd** (365 nm, DMF-d7, 2h, rt) to generate *cis*-**5-Pd**. Absorptions with an asterisk belong to *cis*-**5-Pd**.

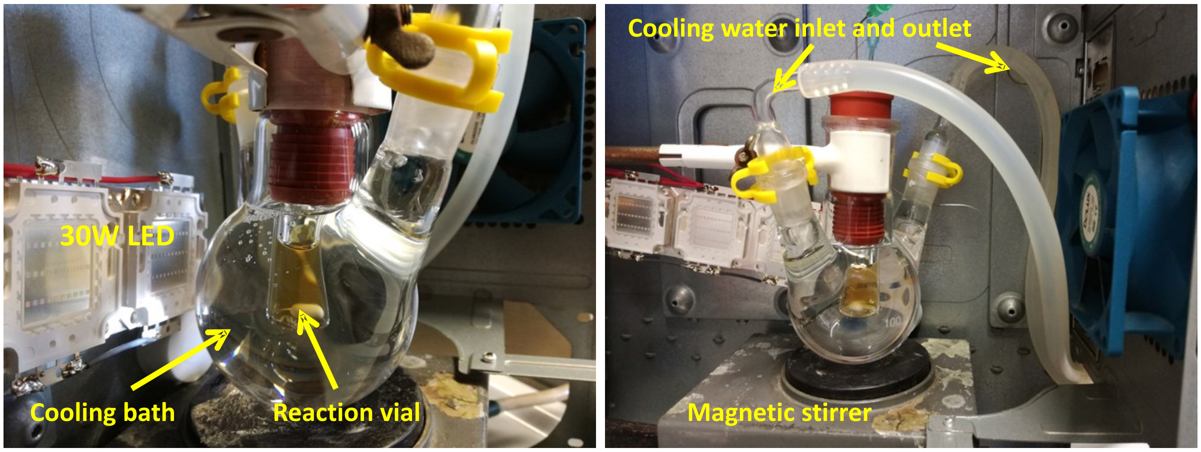

Figure S2. Inside view of the multi-wavelength 30 W LED photoreactor equipped with a flow-through cooling system. The 365 and 440 nm LEDs were operated at a current of 1.0 A supported by a Voltcraft PPS-11603 power supply. Reaction vials were cooled in a constant-flow water bath tempered to 23 +/- 0.5°C.

Suzuki coupling reactions

*Suzuki coupling reaction catalyzed by **5-Pd***

A catalyst suspension was prepared from *trans*-**5-Pd** (2 mg, 3.9 µmol) in DMF/H_2O 1:1 (10 mL) by ultrasonic agitation for 1 min. This suspension was used as the source of catalyst in the following experiments.

*Trans-**5-Pd** catalyzed Suzuki reactions* Phenylboronic acid (1 eq.), aryl halide (1 eq.), potassium carbonate (3 eq.), DMF/H_2O 1:1 (a total of 3 mL including the volume of catalyst suspension) were placed in a vial, sonicated until complete dissolution, then the catalyst suspension containing the necessary amount of Pd was added to the reaction mixture, which was stirred at rt in the absence of light. The progress of the reaction was monitored by GC-MS.

*Cis-**5-Pd** catalyzed Suzuki reactions* In each reaction, for the generation of *cis*-**5-Pd** a suspension of *trans*-**5-Pd** was irradiated with a 365 nm 10 W LED light source for 30 min, and the necessary amount of solution was added to the mixture of reactants (phenylboronic acid (1 eq.), aryl halide

(1 eq.), potassium carbonate (3 eq.), DMF/H_2O 1:1 (a total of 3 mL including the volume of catalyst solution)). In the cases of irradiations with 10 W LED the reaction vials were placed in a rt water cooling bath and continuously irradiated with 365 nm light. However, an active flow-through cooling (23 +/- 0.5°C) of the reaction vial was necessary to exclude the heat effect when the high-power 30 W LED was used. The progress of the reaction was monitored by GC-MS analysis. After completion, the reaction mixture was diluted with EtOAc (20 ml), washed with water (2x20 mL) and brine (10 ml), then dried over anhydrous $MgSO_4$ and the solvent was removed by rotary evaporation. Finally, the crude product was purified by flash column chromatography (SiO_2, *n*-hexane /ethyl acetate 12:1). The ^1H NMR spectrum of the isolated compounds were in agreement with previously reported data.

2. UV-Vis data

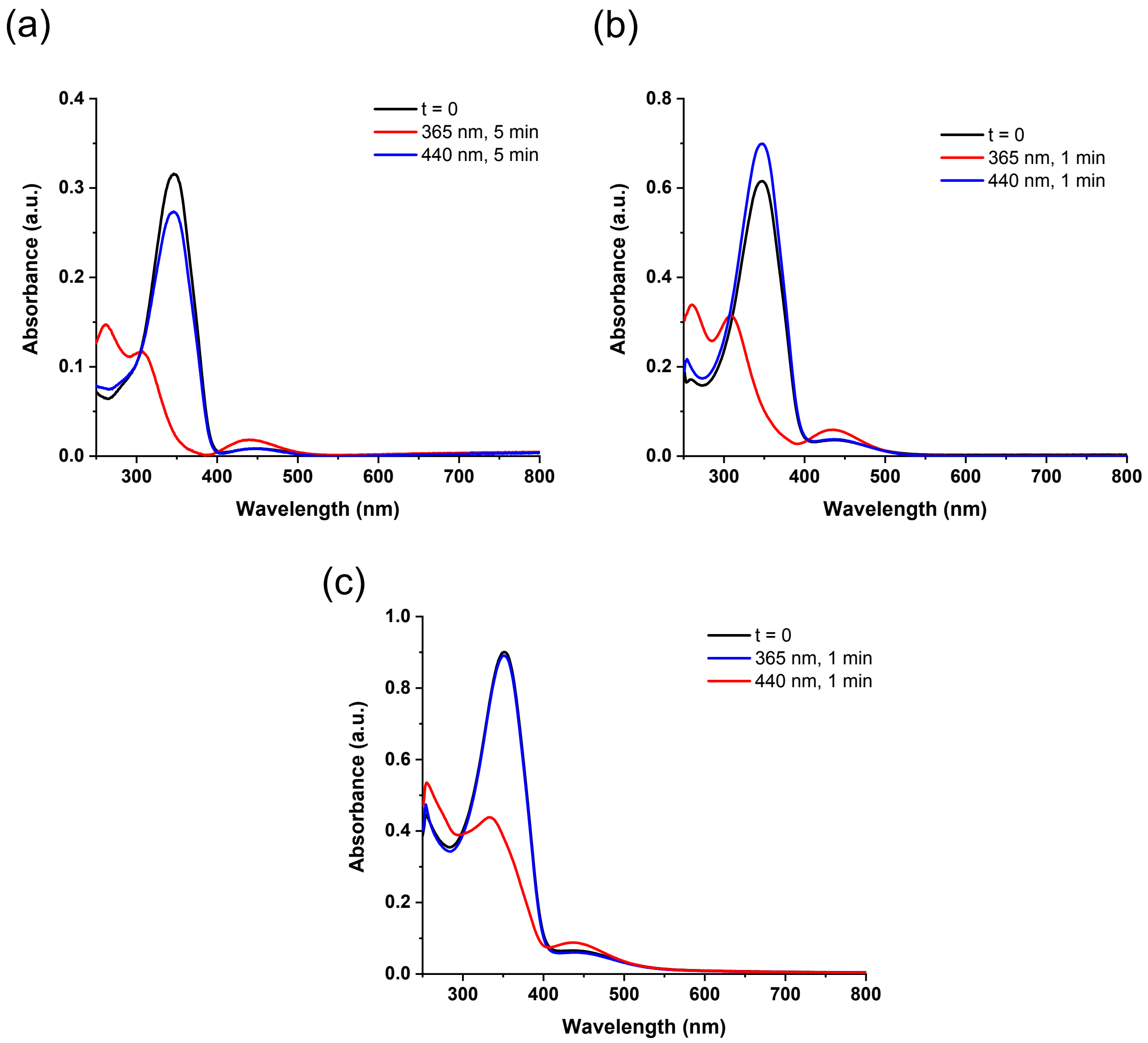

Figure S3. Reversible isomerization of (a) ligand **4** (MeCN, rt), (b) ligand **5** (DMF/H_2O 1:1, rt) and (c) *trans*-**5-Pd** (c = 3 x 10^{-5} M, DMF/H_2O 1:1) followed by UV-Vis spectroscopy.

3. X-Ray Crystallography

Intensity data were collected on a Rigaku RAXIS-RAPID II diffractometer (using graphite monochromator; Mo-Kα radiation, λ = 0.71075Å) at 153 and 143K in case of crystals **4** and **5-Pd**, respectively. Crystals of crystals **5** and **5-Pd** were measured in a loop. Crystal Clear[2] (developed by Rigaku Company) software were used for data collection and refinement. Numerical absorption corrections[3] were applied to the data. The structures were solved by direct methods. Anisotropic full-matrix least-squares refinements were performed on F^2 for all non-hydrogen atoms. Hydrogen atoms bonded to C atoms were placed in calculated positions and refined in a riding-model approximation. The computer programs used for the structure solution, refinement and analysis of the structures were Shelx[4,5], Wingx[6], Platon[7] and Olex2[8]. Program Mercury[9] was used for the graphical representation. Details of crystallographic data, data collection and refinement for crystal crystals **4** and **5-Pd** are collected in Table S2.

Table S2. Summary of crystallographic data, data collections, structure determination and refinement for **4** and **5-Pd**.

Crystals number	**4**	**5-Pd**
CCDC number	2050358	2050357
Empirical formula	C20H$_{16}$N$_6$	$C_{19}H_{22}Cl_2N_6Pd$
Formula weight	340.39	511.72
Temperature (K)	153(2)	143(2)
Radiation and wavelength	Cu-Kα, λ =1.54187Å	Mo-Kα, λ =0.71075Å
Crystal system	triclinic	monoclinic
Space group	*P* -1	*P c*
Unit cell dimensions	*a* =5.8998(2)Å	*a* =12.8830(7)Å
	b =9.3629(3)Å	*b* =8.8827(4)Å
	c =15.9508(6)Å	*c* =9.6095(5)Å
	α =73.354(5)°	α =90°
	β =81.870(6)°	β =110.352(8)°
	γ =81.835(6)°	γ =90°
Volume	830.90(6)Å^3	1031.02(10)Å^3
Z,Z'	2,1	2,1
Density (calculated)	1.361 Mg/m^3	1.648 Mg/m^3
Absorption coefficient, μ	0.685 mm^{-1}	1.177 mm^{-1}
F(000)	356	516
Crystal colour	orange	redish
Crystal description	chunk	platelet
Crystal size (mm)	0.40 x 0.40 x 0.13	0.500x 0.25 x 0.02
Absorption correction	numerical	numerical
Max. and min. transmission	0.834433 and 0.947750	0.772776 and 0.960418
θ–range for data collection	4.959 ≤ θ ≤ 68.230°	3.221 ≤ θ ≤ 26.368°
Index ranges	-7 ≤ *h* ≤7;	-16 ≤ *h* ≤16;

	-11≤ k ≤11;	-11≤ k ≤11;
	-19 ≤ l ≤18	-12 ≤ l ≤12
Reflections collected	17869	41991
Completeness to 2θ	0.983	0.998
Absolute structure parameter		0.032(9)
Friedel coverage		0.993
Friedel fraction max.		0.997
Friedel fraction full		0.997
Independent reflections	2979 [R(int) =0.0475]	4202 [R(int) =0.0304]
Reflections I>2σ(I)	2720	4187
Data / restraints / parameters	2979 /0 /240	4202 /2 /254
Goodness-of-fit on F2	1.105	1.110
Final R indices [I>2σ(I)]	R_1 =0.0432	R_1 =0.0183
	wR^2 =0.1054	wR^2 =0.0374
R indices (all data)	R_1 =0.0484	R_1 =0.0184
	wR^2 =0.1081	wR^2 =0.0375
Max. and mean shift/esd	0.000;0.000	0.001;0.000
Largest diff. peak and hole	0.16;-0.15 e.Å^{-3}	0.34;-0.38 e.Å^{-3}

Crystal data for **4***:* $C_{20}H_{16}N_6$, *Fwt.*: 340.39, orange, chunk, size: 0.400 x 0.400 x 0.130 mm, triclinic, space group P -1, a = 5.8998(2)Å, b = 9.3629(3)Å, c = 15.9508(6)Å, α = 73.354(5)°, β = 81.870(6)°, γ = 81.835(6)°, V = 830.90(6)Å^3, T = 153(2)K, Z= 2, Z'=1, F(000) = 356, D_x = 1.361 Mg/m^3, μ 0.685mm^{-1}.

A crystal of ligand **4** was mounted on a fiber. Cell parameters were determined by least-squares using 16531 (4.96 ≤ θ ≤ 68.31°) reflections.

Intensity data were collected on a Rigaku RAXIS-RAPID II diffractometer (monochromator; Cu-Kα radiation, λ = 1.54187Å) at 153(2) K in the range 4.959 ≤ θ ≤ 68.230. A total of 17869 reflections were collected of which 2979 were unique [R(int) = 0.0475, $R(\sigma)$ = 0.0301]; intensities of 2720 reflections were greater than 2σ(I). Completeness to θ = 0.980.

A numerical absorption correction was applied to the data (the minimum and maximum transmission factors were 0.834433 and 0.947750).

The structure was solved by direct methods (and subsequent difference syntheses).

Anisotropic full-matrix least-squares refinement on F^2 for all non-hydrogen atoms yielded R_1 = 0.0432 and wR^2 = 0.1054for 1332 [I>2σ(I)] and R_1 = 0.0484 and wR^2 = 0.1081 for all (2979) intensity data, (number of parameters = 240, goodness-of-fit = 1.105, the maximum and mean shift/esd is 0.000 and 0.000).

The maximum and minimum residual electron density in the final difference map was 0.163 and -0.150 e.Å^{-3}.

The weighting scheme applied was $w = 1/[\sigma^2(F_o^2)+(0.03720.3023P)^2+0.3023P]$ where $P = (F_o^2+2F_c^2)/3$.

Hydrogen atomic positions were calculated from assumed geometries. Hydrogen atoms were included in structure factor calculations but they were not refined. The isotropic displacement parameters of the hydrogen atoms were approximated from the U(eq) value of the atom they were bonded to.

Crystal data for **5-Pd***:* $C_{19}H_{22}Cl_2N_6Pd$, *Fwt.*: 511.72, redish, platelet, size: 0.500 x 0.250 x 0.020 mm, monoclinic, space group *P* c, a = 12.8830(7)Å, b = 8.8827(4)Å, c = 9.6095(5)Å, α = 90°, β = 110.352(8)°, γ = 90°, V = 1031.02(10)Å^3, T = 143(2)K, Z= 2, Z'=1, F(000) = 516, D_x = 1.648 Mg/m^3, μ 1.177mm^{-1}.

A crystal of **5-Pd** was mounted on a fiber. Cell parameters were determined by least-squares using 46143 (3.22 ≤ θ ≤ 27.46°) reflections.

Intensity data were collected on a Rigaku RAXIS-RAPID II diffractometer (monochromator; Mo-*K*α radiation, λ = 0.71075Å) at 143(2) K in the range 3.221 ≤ θ ≤ 26.368. A total of 41991 reflections were collected of which 4202were unique [R(int) = 0.0304, $R(\sigma)$ = 0.0145]; intensities of 4187 reflections were greater than 2σ(I). Completeness to θ = 0.999.

A numerical absorption correction was applied to the data (the minimum and maximum transmission factors were 0.772776 and 0.960418).

The structure was solved by direct methods (and subsequent difference syntheses).

Anisotropic full-matrix least-squares refinement on F^2 for all non-hydrogen atoms yielded R_1 = 0.0183 and wR^2 = 0.0374for 1332 [I>2σ(I)] and R_1 = 0.0184 and wR^2 = 0.0375 for all (4202) intensity data, (number of parameters = 254, goodness-of-fit = 1.110, the maximum and mean shift/esd is 0.001 and 0.000). The absolute structure parameter is 0.032(9). (Friedel coverage: 0.993, Friedel fraction max.: 0.997, Friedel fraction full: 0.997).

The maximum and minimum residual electron density in the final difference map was 0.34 and -0.38 eÅ^{-3}.

The weighting scheme applied was $w = 1/[\sigma^2(F_o^2)+(0.01780.3979P)^2+0.3979P]$ where $P = (F_o^2+2F_c^2)/3$.

Hydrogen atomic positions were calculated from assumed geometries. Hydrogen atoms were included in structure factor calculations but they were not refined. The isotropic displacement

parameters of the hydrogen atoms were approximated from the U(eq) value of the atom they were bonded to.

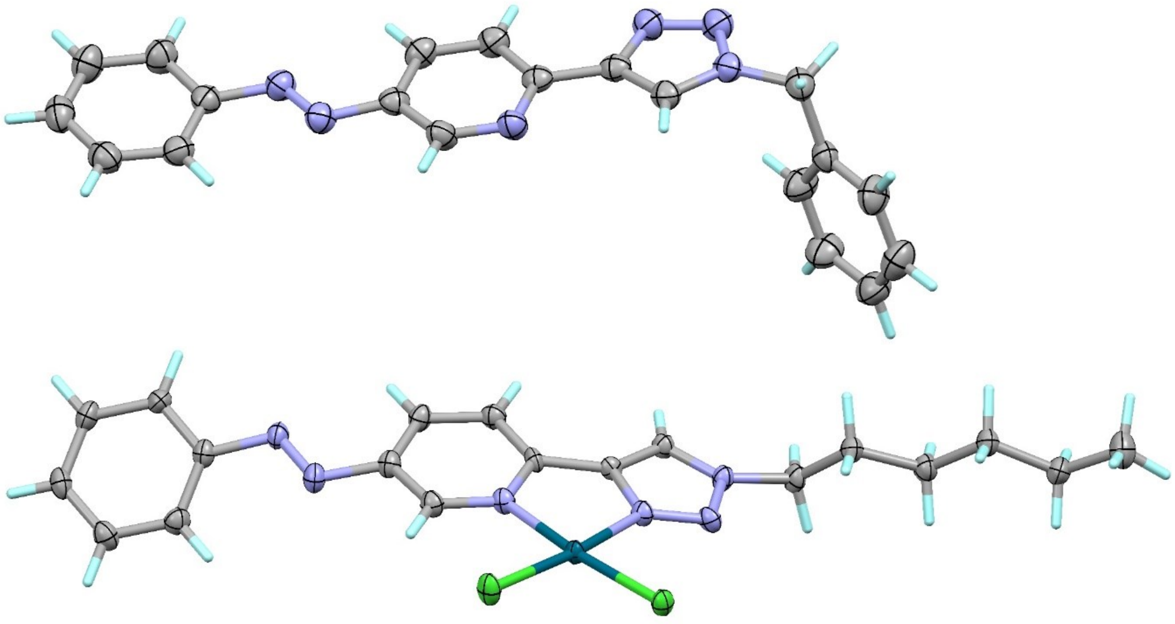

Figure S4. Ortep styles diagram of the asymmetric unit in **4** (top) and **5-Pd** (bottom).

Direction	**4**	**5-Pd**
***a* axis**	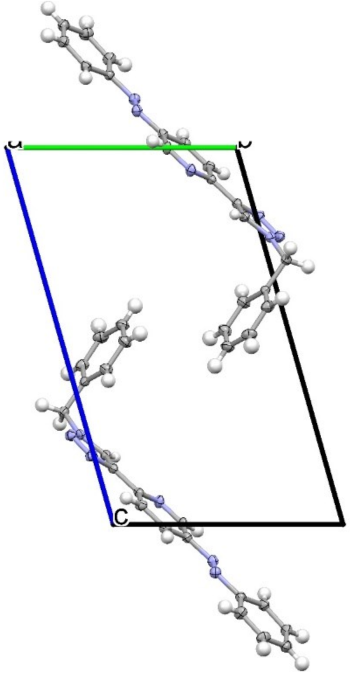	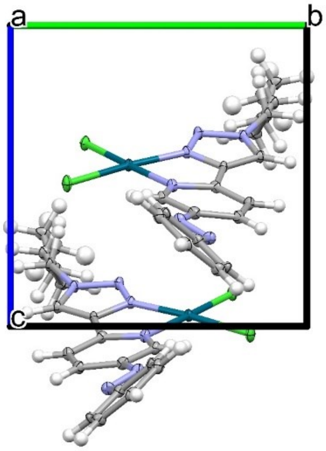
***b* axis**	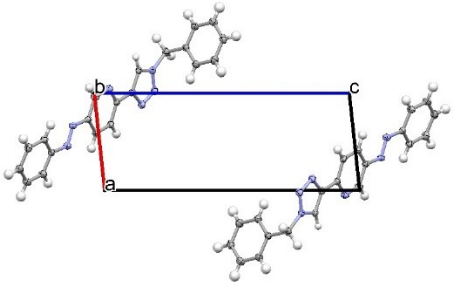	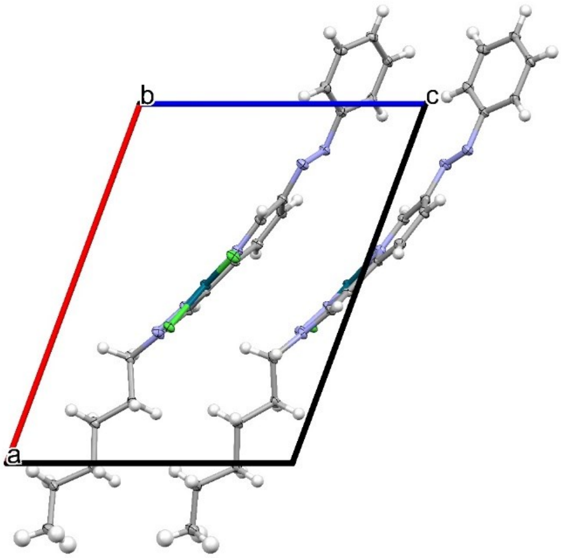
***c* axis**	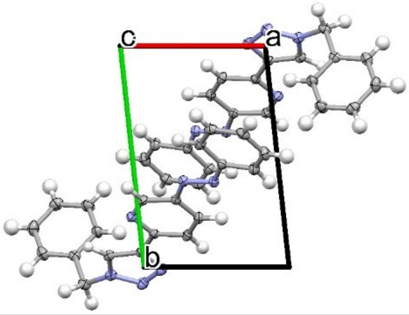	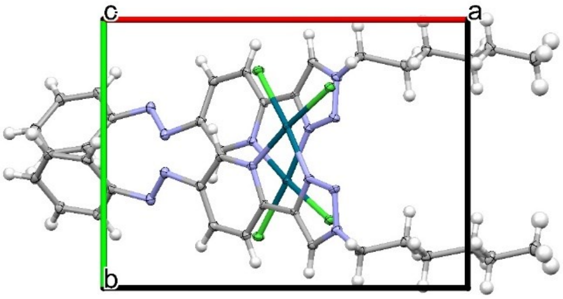

Figure S5. Comparison of the packing motifs in the different crystals of **4** and **5-Pd**.

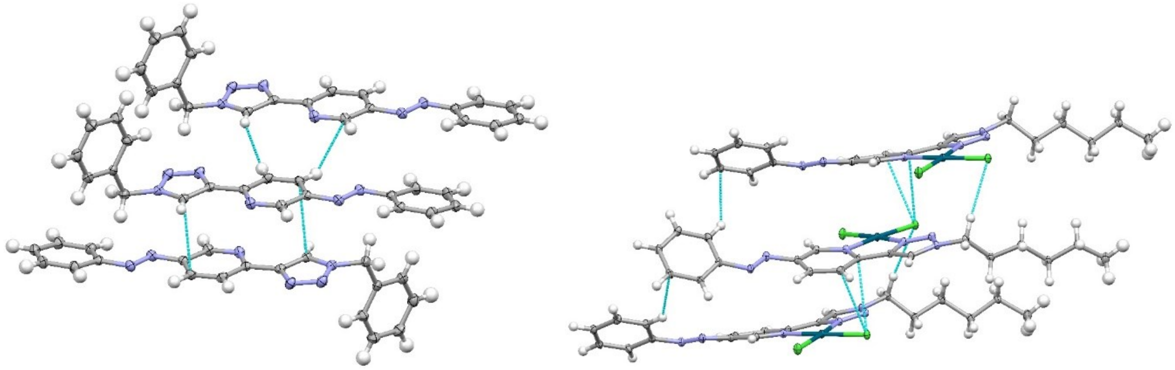

Figure S6. Short contacts within the molecules in the **4** (left) and **5-Pd** (right) crystal lattices.

5. NMR Spectra

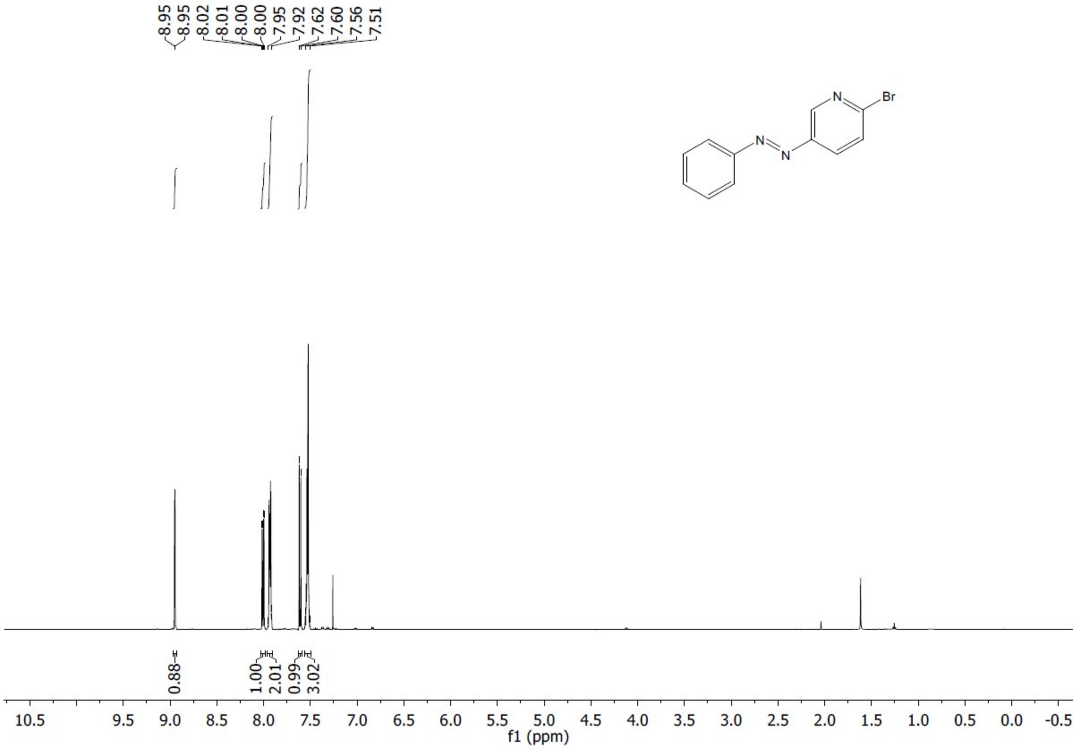

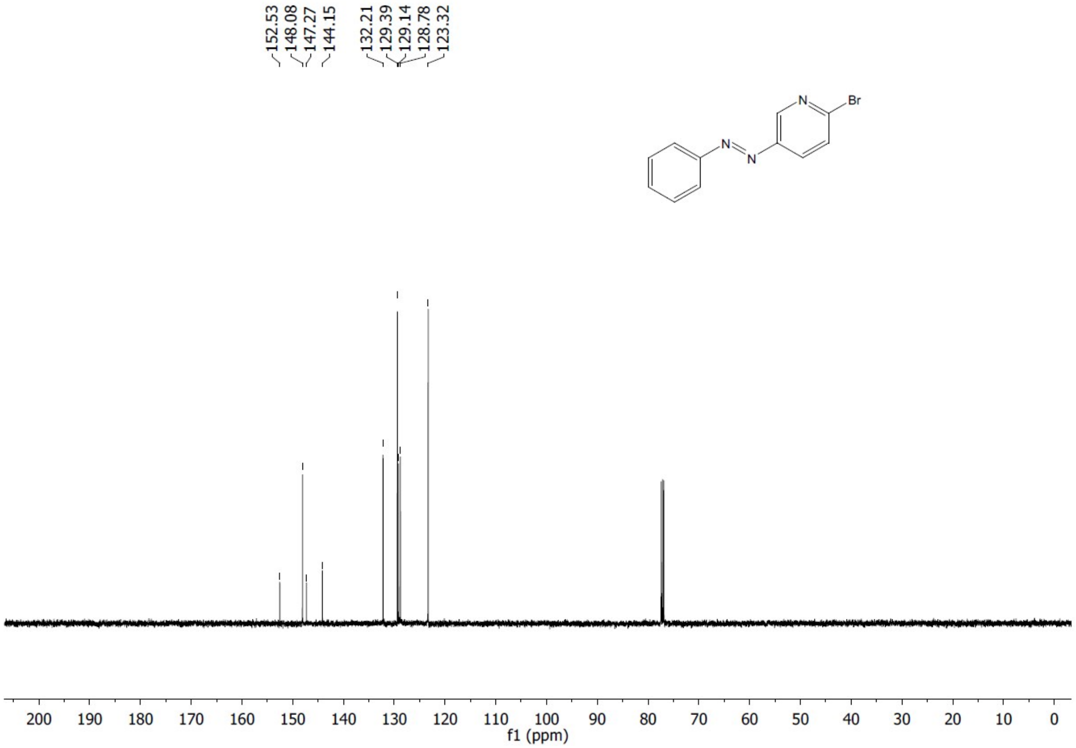

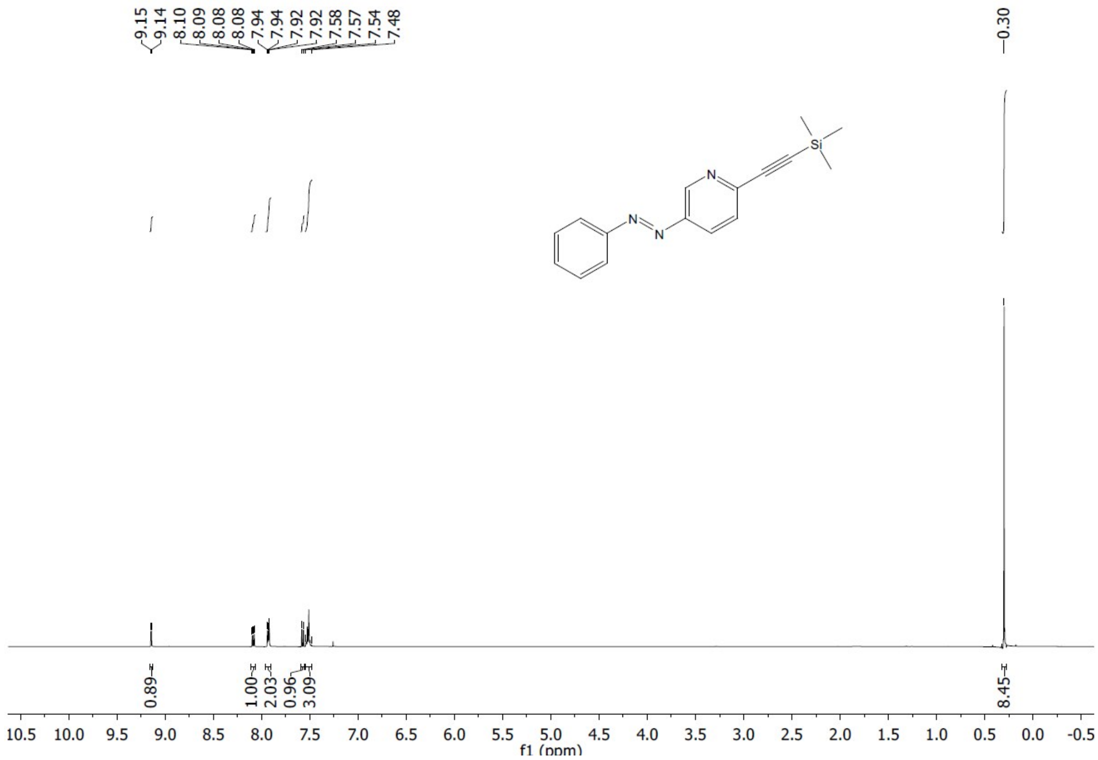
9.15
9.14
8.10
8.09
8.08
8.08
7.94
7.94
7.92
7.92
7.58
7.57
7.54
7.48
0.30
N
N
N
Si
0.89
1.00
2.03
0.96
3.09
8.45
10.5 10.0 9.5 9.0 8.5 8.0 7.5 7.0 6.5 6.0 5.5 5.0 4.5 4.0 3.5 3.0 2.5 2.0 1.5 1.0 0.5 0.0 -0.5
f1 (ppm)

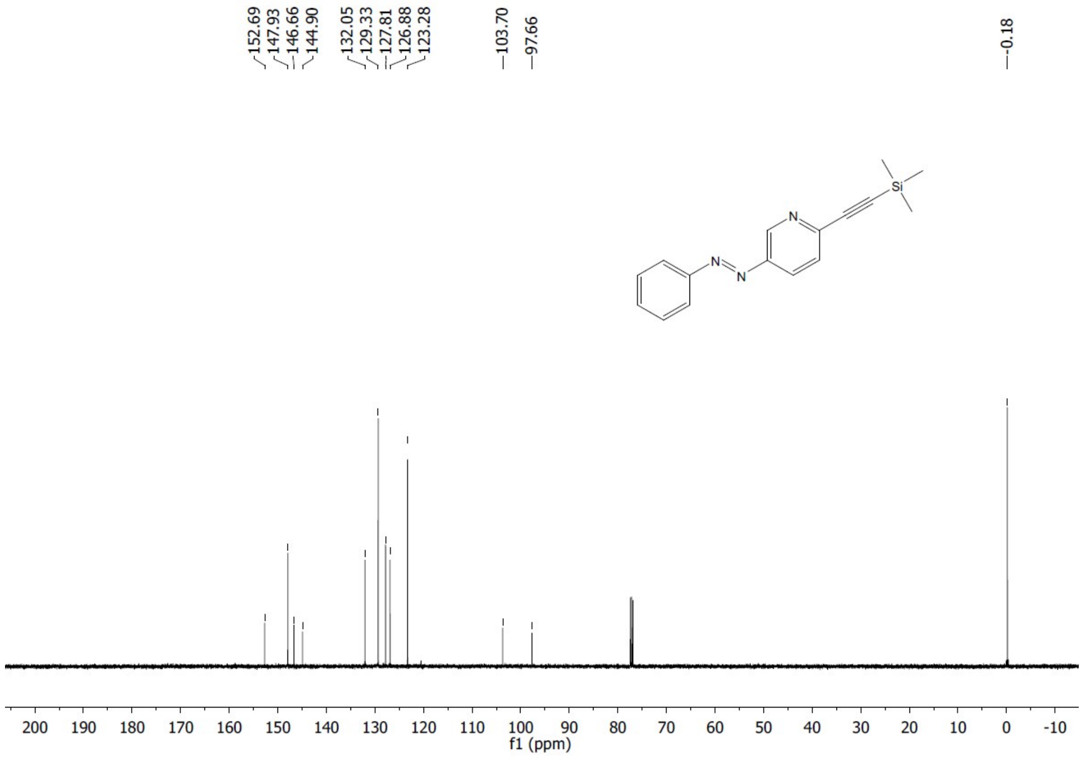
152.69
147.93
146.66
144.90
132.05
129.33
127.81
126.88
123.28
103.70
97.66
-0.18
N
N
N
Si
200 190 180 170 160 150 140 130 120 110 100 90 80 70 60 50 40 30 20 10 0 -10
f1 (ppm)

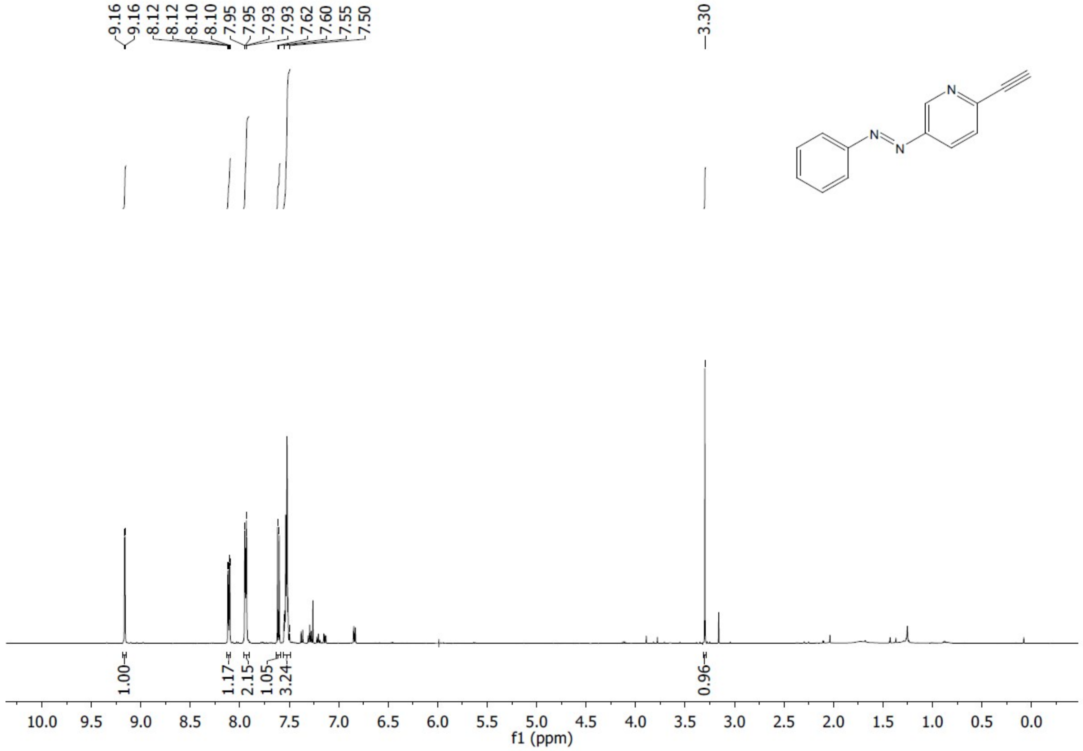
9.16
9.16
8.12
8.12
8.10
8.10
7.95
7.95
7.93
7.93
7.62
7.60
7.55
7.50
3.30
N
N
N
1.00
1.17
2.15
1.05
3.24
0.96
10.0 9.5 9.0 8.5 8.0 7.5 7.0 6.5 6.0 5.5 5.0 4.5 4.0 3.5 3.0 2.5 2.0 1.5 1.0 0.5 0.0
f1 (ppm)

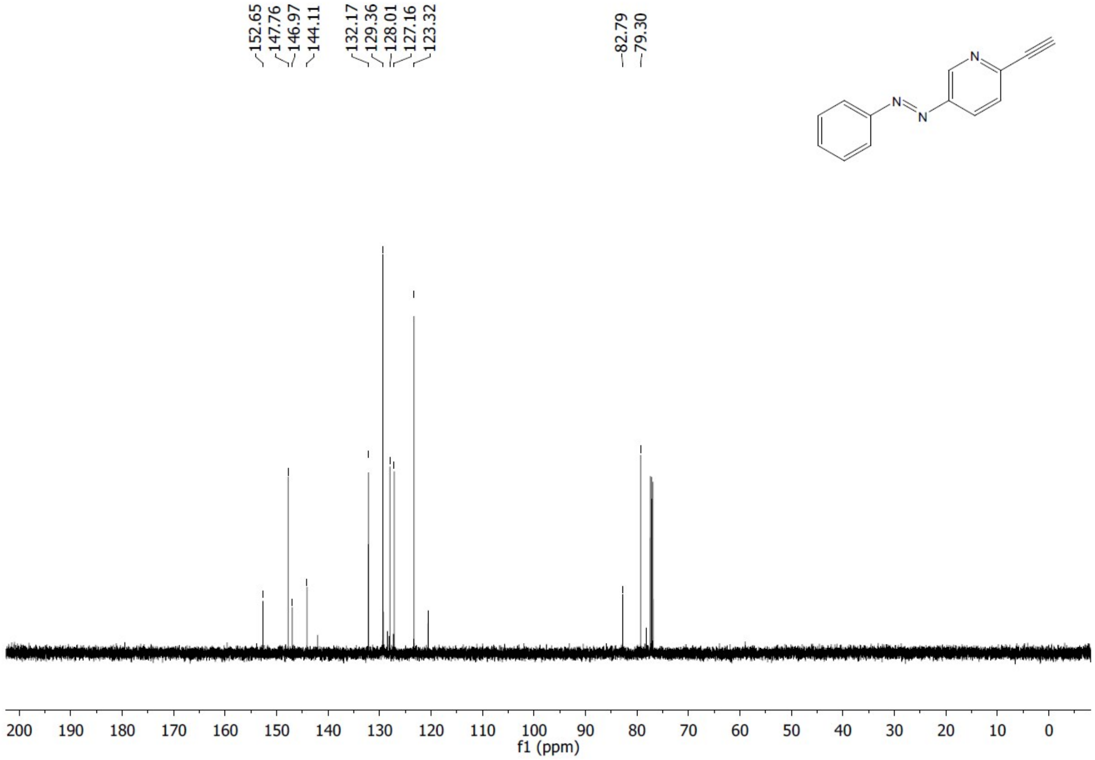
152.65
147.76
146.97
144.11
132.17
129.36
128.01
127.16
123.32
82.79
79.30
N
N
N
200 190 180 170 160 150 140 130 120 110 100 90 80 70 60 50 40 30 20 10 0
f1 (ppm)

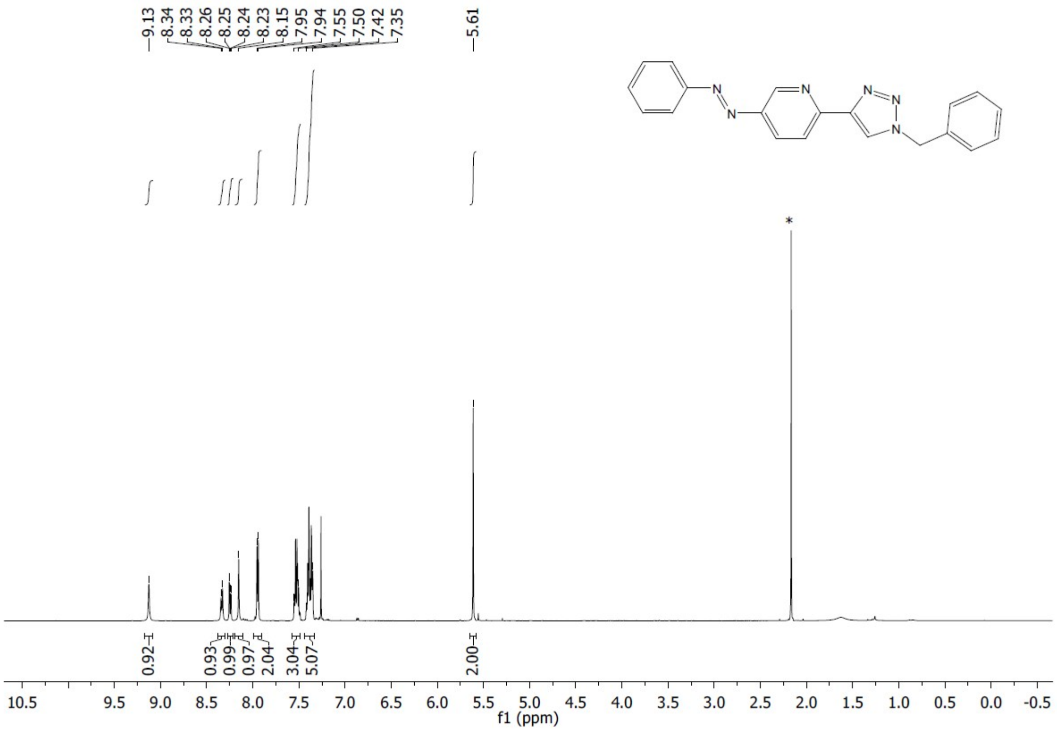
9.13
8.34
8.33
8.26
8.25
8.24
8.23
8.15
7.95
7.94
7.55
7.50
7.42
7.35
5.61
*
0.92
0.93
0.99
0.97
2.04
3.04
5.07
2.00
10.5 9.5 9.0 8.5 8.0 7.5 7.0 6.5 6.0 5.5 5.0 4.5 4.0 3.5 3.0 2.5 2.0 1.5 1.0 0.5 0.0 -0.5
f1 (ppm)

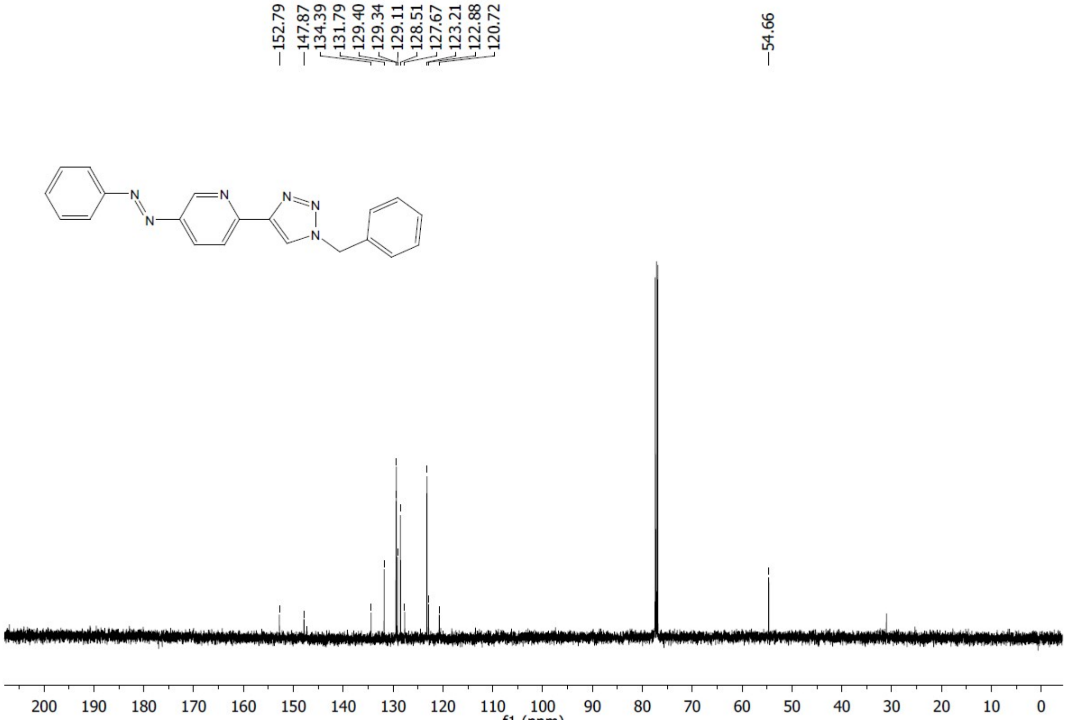
152.79
147.87
134.39
131.79
129.40
129.34
129.11
128.51
127.67
123.21
122.88
120.72
54.66
200 190 180 170 160 150 140 130 120 110 100 90 80 70 60 50 40 30 20 10 0

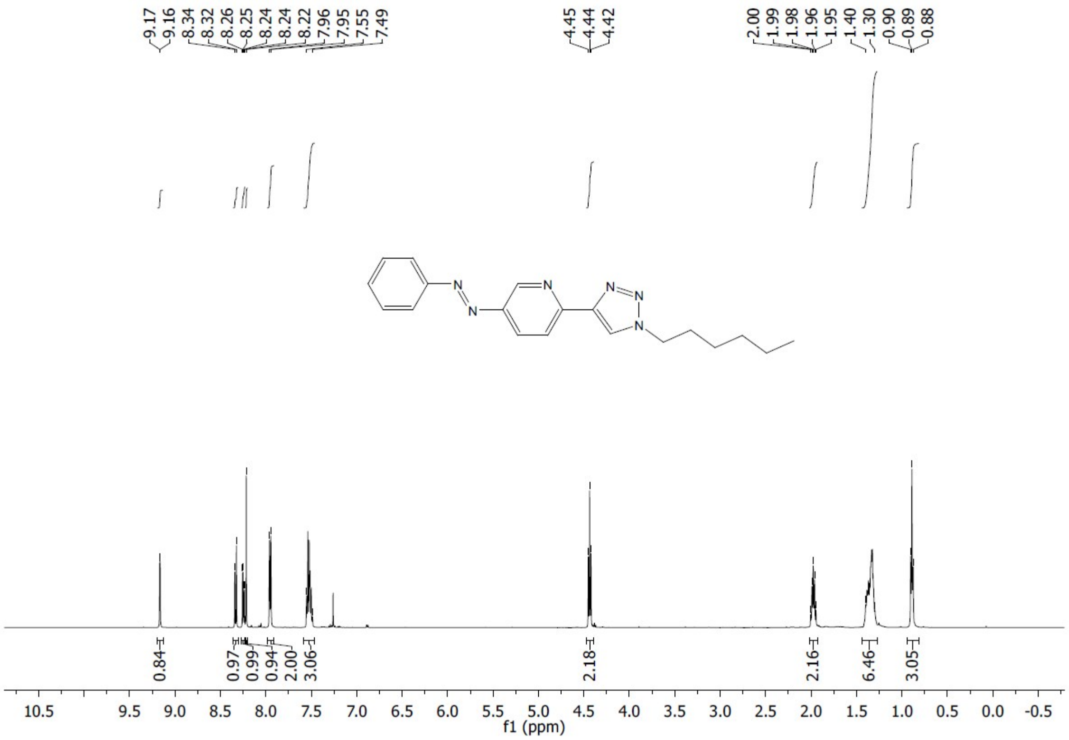
9.17
9.16
8.34
8.32
8.26
8.25
8.24
8.24
8.22
7.96
7.95
7.55
7.49
4.45
4.44
4.42
2.00
1.99
1.98
1.96
1.95
1.40
1.30
0.90
0.89
0.88
N
N
N
N
N
N
0.84
0.97
0.99
0.94
2.00
3.06
2.18
2.16
6.46
3.05
10.5 9.5 9.0 8.5 8.0 7.5 7.0 6.5 6.0 5.5 5.0 4.5 4.0 3.5 3.0 2.5 2.0 1.5 1.0 0.5 0.0 -0.5
f1 (ppm)

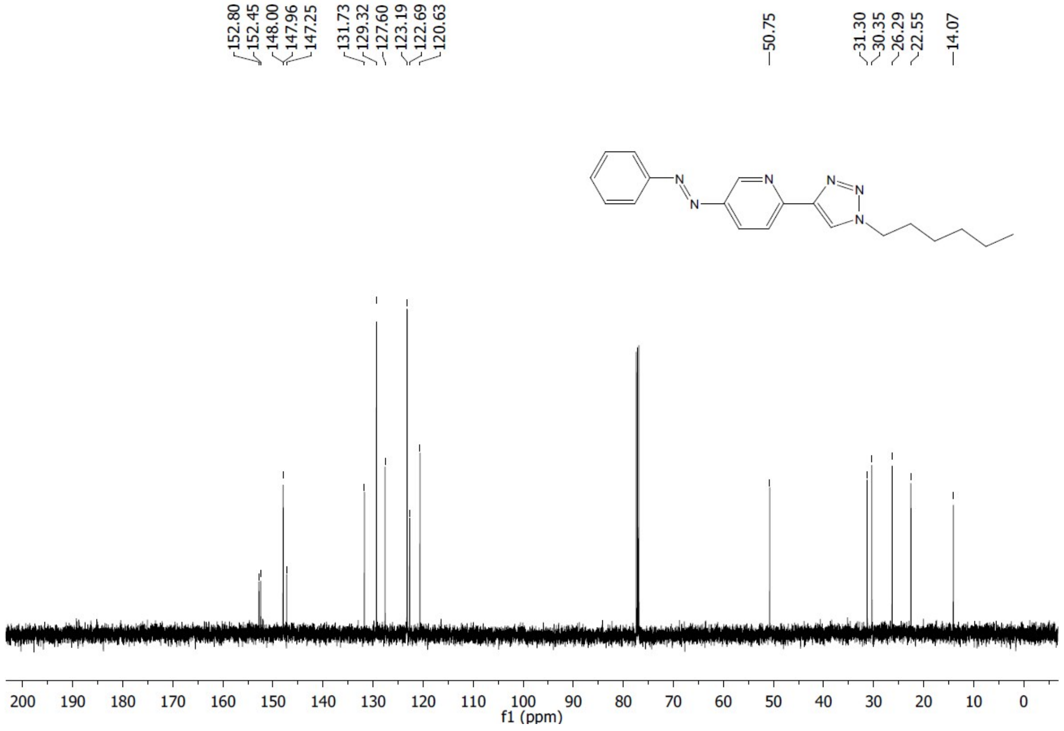
152.80
152.45
148.00
147.96
147.25
131.73
129.32
127.60
123.19
122.69
120.63
50.75
31.30
30.35
26.29
22.55
14.07
N
N
N
N
N
N
200 190 180 170 160 150 140 130 120 110 100 90 80 70 60 50 40 30 20 10 0
f1 (ppm)

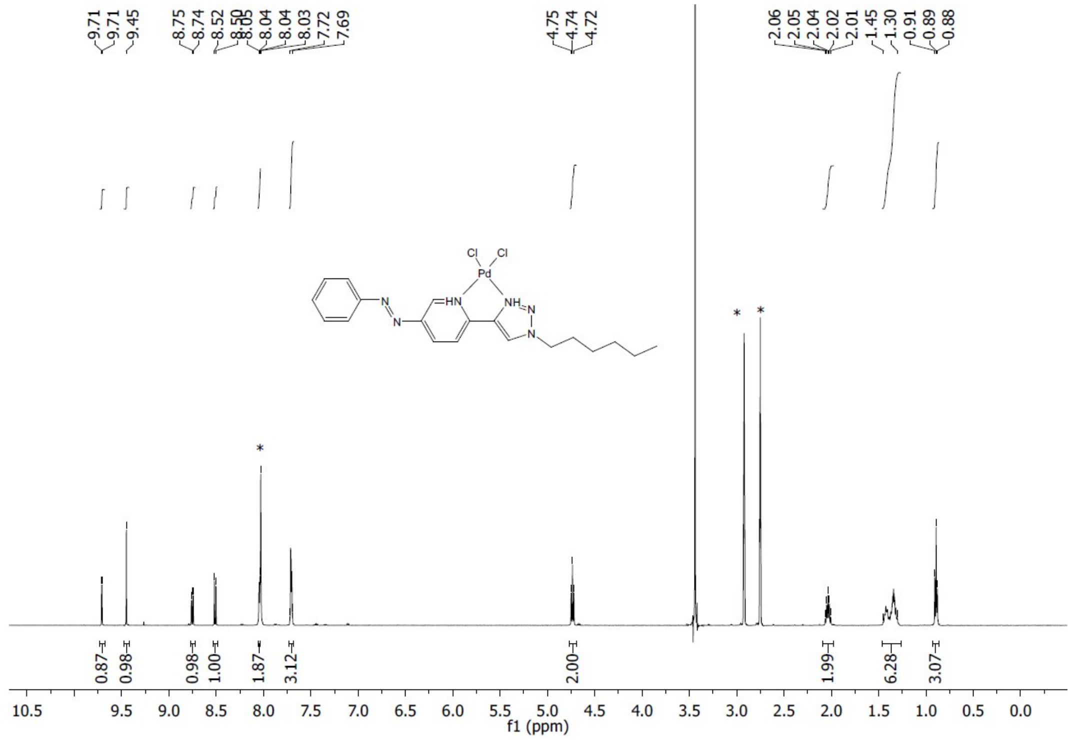
9.71
9.71
9.45
8.75
8.74
8.52
8.50
8.05
8.04
8.04
8.03
7.72
7.69
4.75
4.74
4.72
2.06
2.05
2.04
2.02
2.01
1.45
1.30
0.91
0.89
0.88
Cl Cl
Pd
HN
NH
N
N
N
N
N
0.87
0.98
0.98
1.00
1.87
3.12
2.00
1.99
6.28
3.07
*
*
*
f1 (ppm)

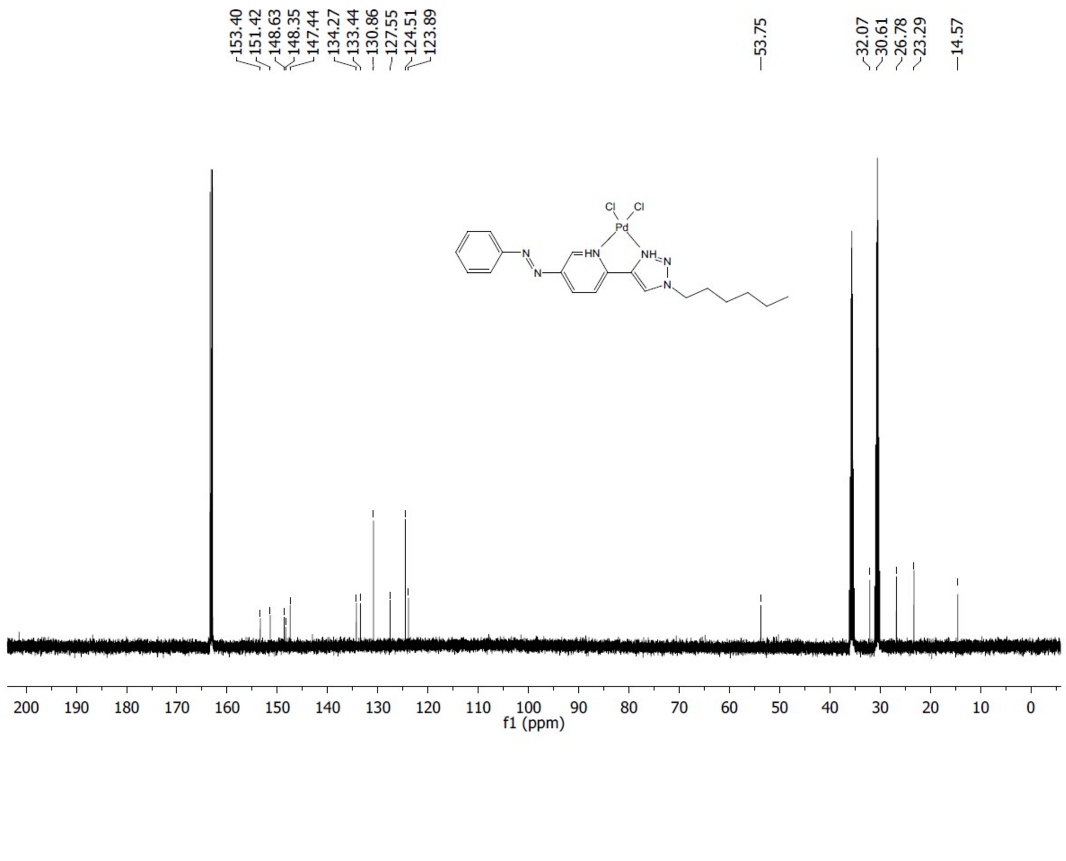
153.40
151.42
148.63
148.35
147.44
134.27
133.44
130.86
127.55
124.51
123.89
53.75
32.07
30.61
26.78
23.29
14.57
Cl Cl
Pd
HN
NH
N
N
N
N
N
f1 (ppm)

6. References

1. S. O. Schmidt, H. Naggert, A. Buchholz, H. Brandenburg, A. Bannwarth, W. Plass, F. Tuczek, *Eur. J. Inorg. Chem.*, 2016, 2175–2186.
2. CrystalClear SM 1.4.0 Rigaku/MSC Inc., 2008.
3. NUMABS: T. Higashi, (1998), rev. 2002. (Rigaku/MSC Inc.)
4. G. M. Sheldrick, *Acta Cryst.*, 2008, **A64**, 112–122.
5. G. M. Sheldrick, *Acta Cryst.*, 2015, **C71**, 3–8.
6. L. J. Farrugia, *J. Appl. Cryst.*, 2012, **45**, 849–854.
7. A. L. Spek, *Acta Cryst.*, 2009, **D65**, 148.
8. O. V. Dolomanov, L. J. Bourhis, R. J. Gildea, J. A. K. Howard, H. Puschmann, *J. Appl. Cryst.*, 2009, **42**, 339–341.
9. C. F. Macrae, P. R. Edgington, P. McCabe, E. Pidcock, G. P. Shields, R. Taylor, M. Towler, J. van de Streek, *J. Appl. Cryst.*, 2006, **39**, 453–457.
10. D. Schweinfurth, R. Pattacini, S. Strobela, B. Sarkar, *Dalton Trans.*, 2009, 9291–9297.
